# The [*PSI*^+^] yeast prion does not wildly affect proteome composition whereas selective pressure exerted on [*PSI*^+^] cells can promote aneuploidy

**DOI:** 10.1038/s41598-017-07999-8

**Published:** 2017-08-16

**Authors:** Patrick H. W. Chan, Lisa Lee, Erin Kim, Tony Hui, Nikolay Stoynov, Roy Nassar, Michelle Moksa, Dale M. Cameron, Martin Hirst, Joerg Gsponer, Thibault Mayor

**Affiliations:** 1 Department of Biochemistry and Molecular Biology, University of British Columbia, Vancouver, BC Canada; 2 Michael Smith Laboratories, University of British Columbia, Vancouver, BC Canada; 3 Department of Biology, Ursinus College, Pennsylvania, USA

## Abstract

The yeast Sup35 protein is a subunit of the translation termination factor, and its conversion to the [*PSI* ^+^] prion state leads to more translational read-through. Although extensive studies have been done on [*PSI* ^+^], changes at the proteomic level have not been performed exhaustively. We therefore used a SILAC-based quantitative mass spectrometry approach and identified 4187 proteins from both [*psi* ^−^] and [*PSI* ^+^] strains. Surprisingly, there was very little difference between the two proteomes under standard growth conditions. We found however that several [*PSI* ^+^] strains harbored an additional chromosome, such as chromosome I. Albeit, we found no evidence to support that [*PSI* ^+^] induces chromosomal instability (CIN). Instead we hypothesized that the selective pressure applied during the establishment of [*PSI* ^+^]-containing strains could lead to a supernumerary chromosome due to the presence of the *ade1-14* selective marker for translational read-through. We therefore verified that there was no prevalence of disomy among newly generated [*PSI* ^+^] strains in absence of strong selection pressure. We also noticed that low amounts of adenine in media could lead to higher levels of mitochondrial DNA in [*PSI* ^+^] in *ade1-14* cells. Our study has important significance for the establishment and manipulation of yeast strains with the Sup35 prion.

## Introduction

The mammalian prion protein is an infectious element capable of switching from its native fold into a distinct and stable structure, which can then induce a similar conformational change to other mammalian prion proteins^1^. Several proteins in fungi can also induce templated conformational changes, leading to the non-Mendelian inheritance of specific traits^2^. This includes the prominently studied [*PSI* ^+^]. The Sup35 protein in *Saccharomyces cerevisiae*, also known as the eukaryotic translation release factor 3 (eRF3), is found to exist either in the [*psi* ^−^] (non-prion) or [*PSI* ^+^] (prion) forms^3^. Sup35 works in combination with Sup45, the eukaryotic release factor 1 (eRF1), to terminate translation at stop codons by causing the release of polypeptide chains^4^. This translation function is carried out by the C-terminal domain of Sup35, while its N-terminal domain has been proposed to be involved in signalling mRNA decay in conjunction with translation termination^5^.

Interestingly, the N-terminal domain (prion domain) is abnormally rich in glutamine-asparagine (Q-N; residues ~1–40) and oligopeptide repeats (residues ~40–114) which facilitate the formation of amyloid-like aggregates and propagation of [*PSI* ^+^]^6–9^. Translation termination therefore becomes impaired in cells that possess the [*PSI* ^+^] prion as the amount of soluble Sup35 is diminished^10^. Depending on the size and type of aggregates formed however, [*PSI* ^+^] aggregates may retain some functional translation termination activity^11^. Overall since polypeptides may not be consistently released from the ribosome, read-through of stop codons may occur, resulting in the presence of C-terminal extended proteins in the cell.

Depending on the extent of read-through and the biochemical pathways involved, C-terminal extended proteins may be beneficial or detrimental to yeast^12–14^. For many [*PSI* ^+^] strains it was shown that the presence of Sup35 prions either killed the cells or greatly impeded growth^15^. A recent transcriptomic study indicated that a number of genes related to stress pathways were repressed in [*PSI* ^+^] cells compared to [*psi* ^−^]^16^. This suggests that [*PSI* ^+^] yeast may not be able to respond as effectively to stress compared to [*psi* ^−^]. Significantly affected pathways include the oxidative stress response, in addition to other metabolic pathways. However, while [*PSI* ^+^] is found to be deleterious in many circumstances, evolutionary models indicate that [*PSI* ^+^] continues to be maintained in yeast via a bet-hedging strategy^17, 18^. It was proposed that the presence of [*PSI* ^+^] in wild yeast populations could result from the positive selection for prions during rare occasions of extreme or rapidly changing environments, and is hypothesized to accelerate the capacity for evolutionary change by revealing hidden variation present past the stop codons^19^. In this case, the presence of C-terminal extended polypeptides may potentially encode for proteins with enhanced functions in stress pathways. These findings indicate that Sup35 prions may have a detrimental effect on yeast survival in some environments, but continued to be maintained through selective positive pressure in times of rapid environmental change. Hence, a major unresolved question is how the proteome is affected in the presence of [*PSI* ^+^] in order to better determine which pathways may be disrupted or altered in these cells.

Although extensive studies have been done on [*PSI* ^+^], changes in global proteomic levels have not been assessed. Here, we provide the first in-depth proteomic analysis of [*psi* ^−^] and [*PSI* ^+^] using SILAC-based quantitative mass spectrometry using the S288C yeast strain grown in standard growth conditions. Whereas we did not find major differences between both proteomes, we noticed that Chromosome I disomy was prevalent in [*PSI* ^+^] strains that we generated through multiple independent experiments and prion induction methods, most likely due to selection pressure as [*PSI* ^+^] did not induce chromosomal instability. We therefore verified by next generation sequencing (NGS) that there was no prevalence of disomic cells when [*PSI* ^+^] cells were isolated in the absence of strong selective pressure.

## Results

### Generation of [*psi*^−^]_SILAC_ and [*PSI*^+^]_SILAC_ strains

**Figure 1:**
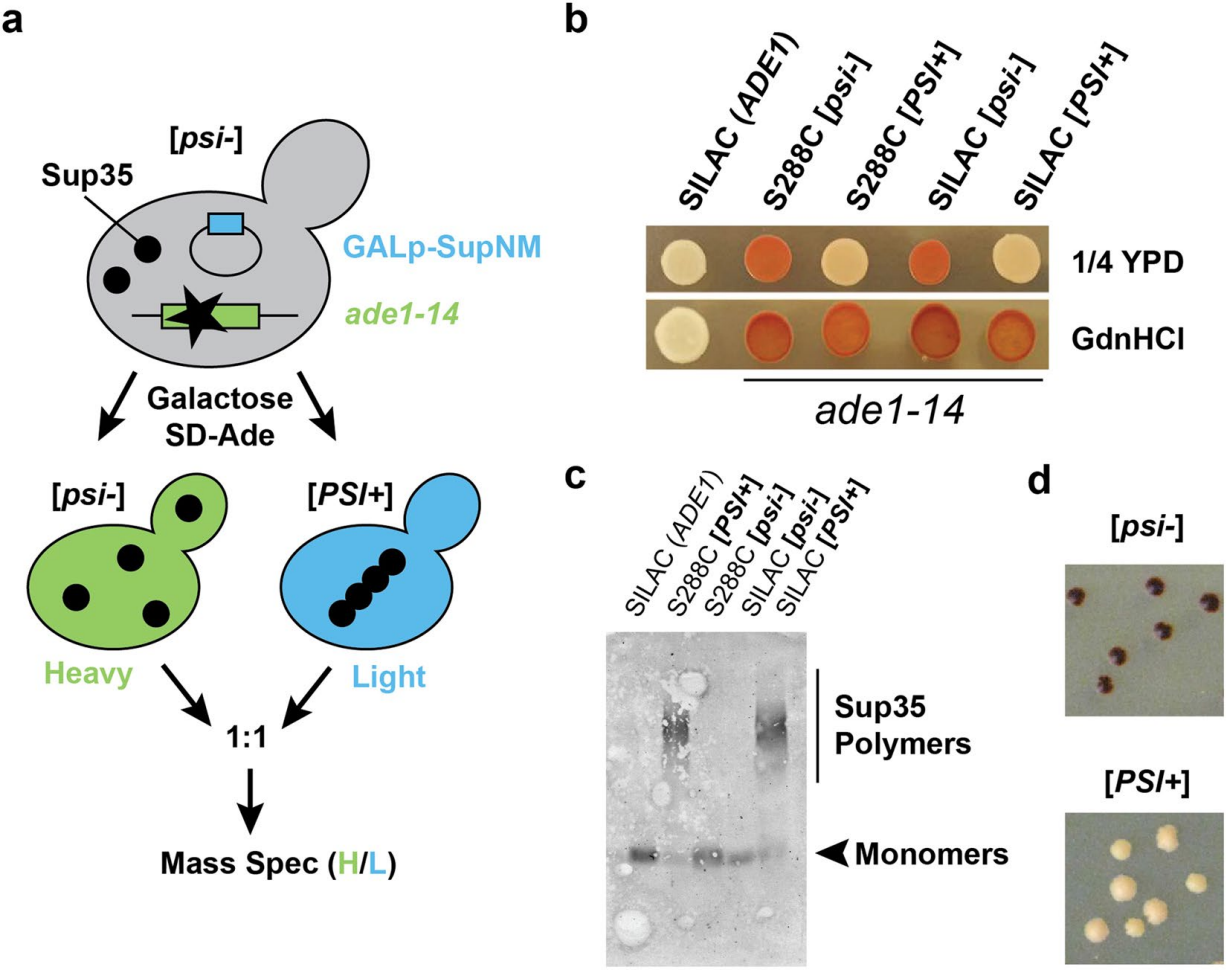
Establishment of the [*psi* ^−^] and [*PSI* ^+^] strains for SILAC analysis. (**a**) Schematic diagram of the SILAC approach. [*PSI* ^+^] cells were generated following the galactose-induced overexpression of exogenous Sup35NM, and selection of *ade1-14* cells in media lacking adenine. (**b**) Indicated strains were grown in the indicated plates. (**c**) SDD-AGE of the lysates of the indicated strains with anti-Sup35 antibodies. (**d**) Colonies from [*PSI* ^+^]_SILAC_ and [*psi* ^−^]_SILAC_ cells that were grown for 5 days on 1/4 YPD plates (see also Fig. S1a).

In order to compare the proteomes from [*psi* ^−^] and [*PSI* ^+^] cells, we employed a SILAC (stable isotope labeling by amino acids in cell culture) approach (Fig. 1a). We first generated the auxotrophic [*psi* ^−^]_SILAC_ strain by mating a S288C (*arg4*Δ/*lys2*Δ) strain with S288C [*PIN* ^+^][*psi* ^−^] cells (presence of the Rnq1 [*PIN* ^+^] prion is required for [*PSI* ^+^] formation)^20^ that carried out the *ade1-14* allele. We then induced the formation of the Sup35 [*PSI* ^+^] prion in [*psi* ^−^]_SILAC_ cells by overexpressing an exogenous Sup35NM fragment under a *GAL* promoter, following selection on SD − Ade plates containing arginine to produce the [*PSI* ^+^]_SILAC_ strain.

We first validated the presence of [*PSI* ^+^] in the newly generated *S*. *cerevisiae* strain through a colour-based assay. In absence of exogenous adenine, the *ade1-14* nonsense mutation results in a non-functional truncated product in [*psi* ^−^] cells and the accumulation of the *P*-ribosylaminoimidazole biosynthetic intermediate, which turns red upon oxidation. Since [*PSI* ^+^] leads to the aggregation of Sup35 and impairment of translation termination, it promotes the translational read-through of *ade1-14* nonsense transcripts and results in expression of sufficient levels of Ade1. While [*psi* ^−^] cells with *ade1-14* turned red after growth on 1/4 YPD (with low concentration of adenine), the [*PSI* ^+^]_SILAC_ strain remained nearly white in these conditions (Fig. 1b). To exclude that *ade1-14* strains were reverted back to *ADE1*, we confirmed that [*PSI* ^+^] was reversed to [*psi* ^−^] in the presence of guanidine hydrochloride (GdnHCl) (Fig. 1b), which impairs Hsp104 and the inheritance of the prion to the daughter cells^21^.

To further confirm the presence of Sup35 amyloid fibrils in the generated strain, we performed semi-denaturing detergent agarose gel electrophoresis (SDD-AGE). While Sup35 remained mostly in its monomeric form in [*psi* ^−^] and [*psi* ^−^]_SILAC_ strains, it migrated with a slower mobility in the [*PSI* ^+^] and [*PSI* ^+^]_SILAC_ strains, due the formation of higher molecular weight species indicative of fibrils (Fig. 1c). We then confirmed that the prion was stably maintained by overgrowing the colonies. While colonies from the [*psi* ^−^]_SILAC_ strain were smaller and dark red, the colonies from the [*PSI* ^+^]_SILAC_ strain were larger and remained pink to light pink after 5 days of growth (Figs 1d and S1a). Importantly, no section of the colonies reverted to a darker red coloration that would be indicative of a loss of [*PSI* ^+^]. We further confirmed stability of [*PSI* ^+^] in our strains by conducting, with some modifications, a previously published assay in which thermal stress was found to partially cure weak [*PSI* ^+^] but not stronger [*PSI* ^+^] strains, resulting in darker red sectors indicative of [*psi* ^−^]^22^. [*PSI* ^+^] strains initially grown at 30 °C were exposed to thermal stress at 40 °C for 30 min, followed by re-incubation at 30 °C for several days and did not show any red sectoring that would be indicative of prion curing (Fig. S1b). Altogether, these results confirm that [*psi* ^−^]_SILAC_ and [*PSI* ^+^]_SILAC_ strains could represent non-prion and stable prion states suitable for mass spectrometric proteomic analysis and further experimentation.

### Proteomic profiling of [*psi*^−^]_SILAC_ and [*PSI*^+^]_SILAC_

**Figure 2:**
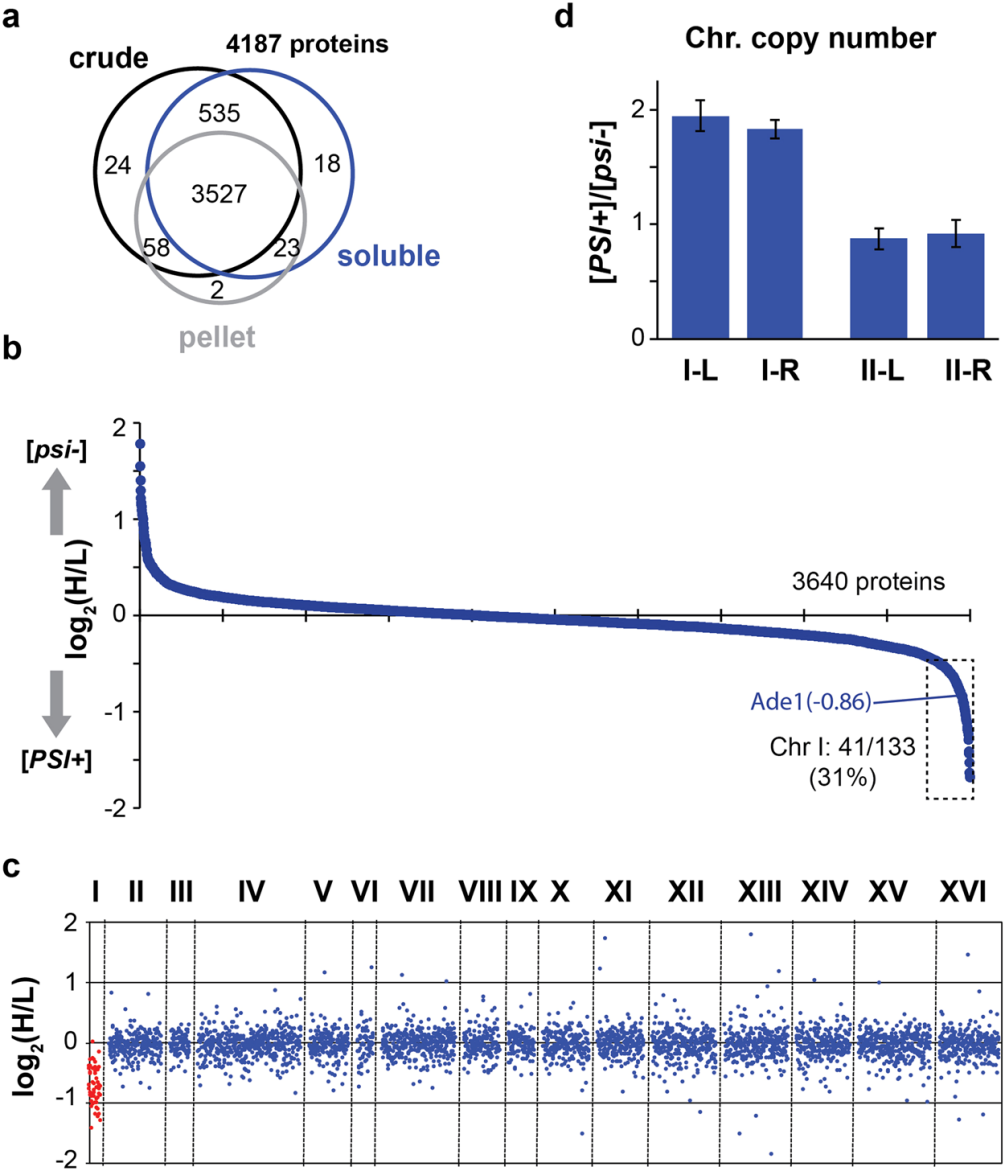
Quantitative mass spectrometry of [*psi* ^−^]_SILAC_ and [*PSI* ^+^]_SILAC_. (**a**) Venn diagrams of proteins identified with ≥2 peptides in the indicated samples. (**b**) Ranked plot of averaged log_2_ ratios (H/L) of proteins quantified from the supernatant samples by MaxQuant. (**c**) Scatter plot of averaged log_2_ ratios (H/L) from the supernatant samples based on the chromosomal locations of corresponding genes. (**d**) Ratios of left (L) and right (R) arms of chromosomes I and II between [*PSI* ^+^]_SILAC_ and [*psi* ^−^]_SILAC_ quantified by qPCR in three replicates (shown with standard deviations).

To quantify potential changes between the proteomes of [*psi* ^−^]_SILAC_ and [*PSI* ^+^]_SILAC_ strains, we labeled cells with either heavy- or light-labeled arginine and lysine residues, respectively (Fig. 1a). We performed the experiment using three biological replicates, and an equal amount of cells were mixed prior to lysis. A fraction of the cells was collected for a crude cell lysis in a denaturing buffer containing urea, and the remaining cells were lysed in a native buffer in order to analyze the soluble and “sedimentable” (pellet) fractions. Cryo-grinding method was employed for cell lysis to avoid foaming and sample heating that are typically induced by other methods. Tryptic peptides were fractionated with basic reverse phase chromatography to generate 96 fractions and every 8 fractions from each sample were combined for liquid chromatography coupled with tandem mass spectrometry (LC-MS) analysis. With the exception of one pellet sample for which there was a poor LC-MS analysis, all the other eight sample spectra were then analyzed with Andromeda using the MaxQuant platform. A total of 4187 yeast proteins (crude: 4144; soluble: 4103; pellet: 3312) were identified with at least two peptides (Fig. 2a; Supplementary Data).

From the supernatant, 3640 proteins were quantified by MaxQuant. Strikingly, we did not observe any major change in protein levels between the [*psi* ^−^]_SILAC_ and [*PSI* ^+^]_SILAC_ strains (Fig. 2b), and only 14 and 22 proteins displayed a two-fold change in protein levels (log_2_ (H/L) >1 and log_2_ (H/L) < −1, respectively). There were however, 121 proteins that were significantly altered between both strains, when considering smaller changes in protein levels (p values < 0.01 and |log_2_ (H/L)| > 0.2 that corresponds to +/−15% changes in protein levels; Fig. S2a). This included Ade1 that was found, as expected, to be more expressed in [*PSI* ^+^] cells (Fig. 2b). Similarly, 34 proteins displayed a two-fold change in protein levels (|log_2_ (H/L)| > 1) among the 2838 and 3722 quantified proteins from the pellet fraction (Fig. S2b; 16 up and 18 down in [*PSI* ^+^] cells) and crude lysate (Fig. S2c; 24 up and 10 down in [*PSI* ^+^] cells), respectively. We quantified levels of 43 proteins among the 75 genes for which less mRNA was associated to ribosomes in [*PSI* ^+^] cells in the study from Namy and colleagues^16^, and only 5 were found to be expressed at lower levels in [*PSI* ^+^] cells (log_2_ (H/L) > 0.5; in this case we lowered the threshold to include more proteins). One possibility is that there are compensatory mechanisms to maintain proteins at higher levels in the cell when these proteins are translated at a lower rate (e.g., by reducing protein turnover). Alternatively, there may be strain-to-strain differences. We did not find any GO term enrichment in any of the samples among the proteins differentially expressed in [*PSI* ^+^] cells (log_2_ (H/L) > 0.5 or <−0.5) or among proteins in the supernatant that were significantly enriched with smaller changes in protein levels (p values < 0.01 and |log_2_ (H/L)| > 0.2). However, among 23 proteins that display lower levels in the pellet fraction of [*PSI* ^+^] cells (log_2_ (H/L) > 0.5) and were quantified in the two samples, there was a significant enrichment for proteins mediating ion transport (p = 0.005) including the hexose transmembrane transport (p = 0.019; Hxt1, Hxt3, Hxt4, Itr1). Hxt3 and Hxt4 were also found expressed at lower levels in the supernatant and crude lysates of [*PSI* ^+^] cells. A [*PSI* ^+^] cell population might have a survival advantage by limiting the intake of sugar when the glucose concentration is low in the environment. These results indicate that although translation termination was impaired in [*PSI* ^+^] cells, there was no major change in proteome composition under the growth conditions used, with a few exceptions.

Previous work by Namy and colleagues^23^ notably identified eight genes in *S*. *cerevisiae* to have enhanced read-through properties (>10-fold stop codon bypass efficiency) compared to mRNA levels. However, they found that only two of those genes, *IMP3* and *BSC4*, displayed increased read-through in a [*PSI* ^+^] in a Sup35-dependent manner. In a subsequent and more recent study, another 68 genes were shown to display an increase of ribosome footprints downstream of the stop codon in [*PSI* ^+^] cells, which might correspond to read-through events^16^. Therefore we aimed to assess if comparable read-through events were occurring in the proteome of our S288C strain. To further evaluate the possible impact of additional read-through events in [*PSI* ^+^] cells, spectra derived from the total cell lysate, supernatant, and pellet samples were also searched against a modified yeast extended database, in which additional amino acids were added to each protein (see Methods). While MaxQuant identified 98 potential peptides that could result from translational read-through of stop codons, only two peptides were retained after manual validation of the spectra and sequences (Fig. S3). These peptides mapped to possible C-terminal extensions of Seg1 (+0) and Dbr1 (+1) but were not quantified. These results indicate that although [*PSI* ^+^] leads to more translation read-through events, proteins with extended C-terminal domains are not readily identified by conventional shotgun proteomics. These data are in agreement with previous studies that showed that [*PSI* ^+^] leads to only 1–3% read-through events^24^.

Interestingly, we noticed that among the 89 proteins for which levels were significantly altered and enriched by at least 15% (p values < 0.01 and log_2_ (H/L) < −0.2) in [*PSI* ^+^]), 22 were encoded by genes on chromosome I (Fig. S2a). Similarly, between 27 and 31% of the proteins with log_2_ (H/L) < −0.5 corresponded to proteins encoded on chromosome I in the three analyzed fractions (Figs 2b, S2b,c). Haploid *S*. *cerevisiae* cells have 16 chromosomes and only 117 genes are located on chromosome I, the shortest one. When we plotted the SILAC ratios based on the chromosomal locations of the corresponding genes, there was a striking alteration of levels of proteins expressed from chromosome I, that were more enriched in [*PSI* ^+^] (Fig. 2c). This result indicates that the [*PSI* ^+^]_SILAC_ strain used in the experiment might have an extra copy of chromosome I. We verified that the selected haploid [*PSI* ^+^]_SILAC_ strain contained an additional copy of chromosome I, but not chromosome II, by qPCR (Fig. 2d). These results demonstrate the selected strain was disomic for chromosome I.

### Identification of multiple aneuploidic [*PSI*^+^]_SILAC_ strains

**Figure 3:**
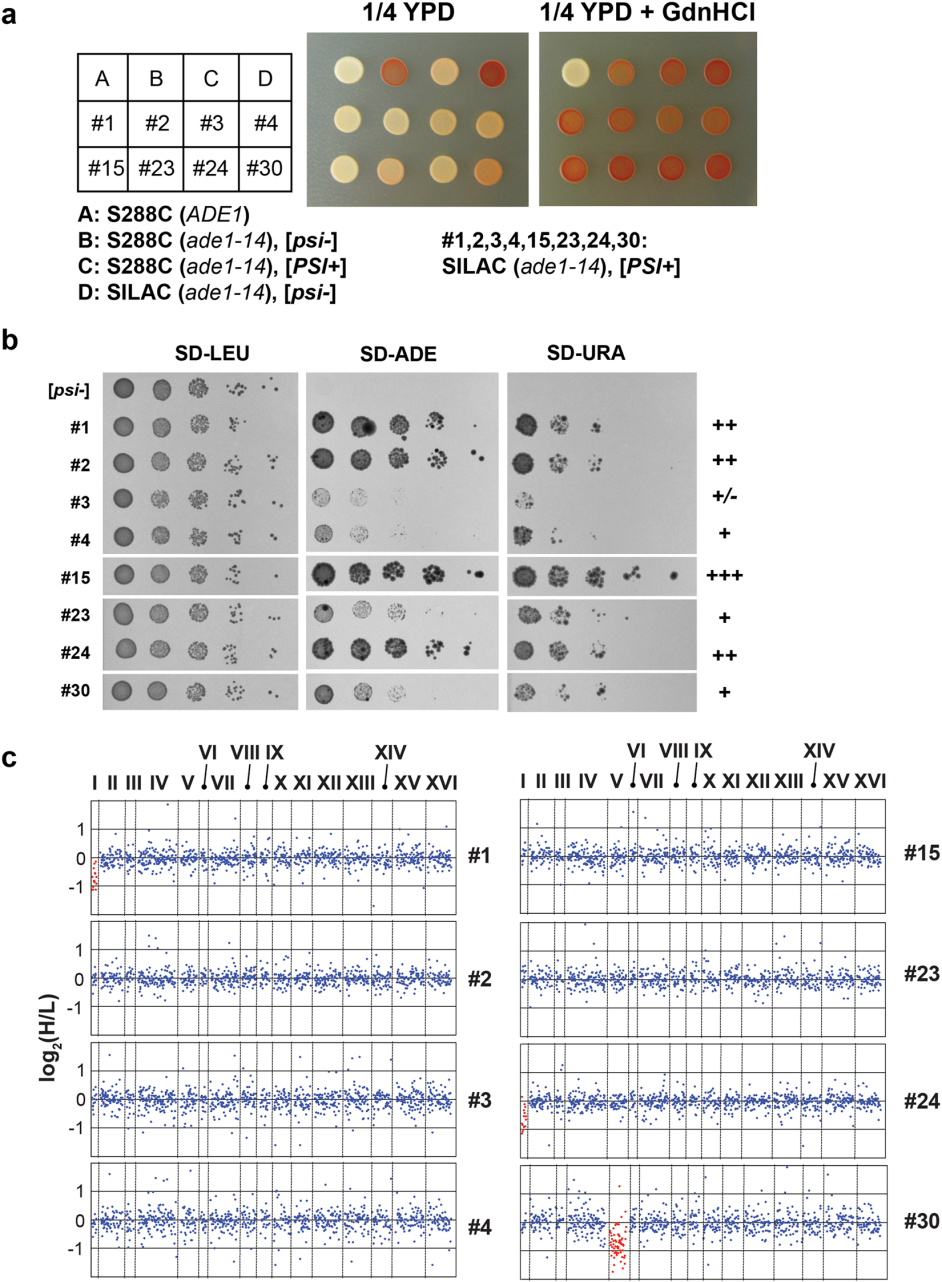
Characterizations of 8 selected [*PSI* ^+^]_SILAC_ strains. (**a**) The indicated strains were grown on the 1/4 YPD plates with and without GdnHCl. (**b**) The indicated [*PSI* ^+^]_SILAC_ strains were grown on the indicated synthetic defined (SD) plates containing 2% dextrose. (**c**) Scatter plots of log_2_ (H/L) of the indicated [*PSI* ^+^]_SILAC_ strains in which proteins were ranked based on their corresponding gene chromosomal locations.

In order to determine whether the disomy in the tested [*PSI* ^+^]_SILAC_ strain (thereafter named [*PSI* ^+^]_SILAC_#1) was due to an unfortunate event during the induction of [*PSI* ^+^], we tested an additional seven [*PSI* ^+^]_SILAC_ strains by mass spectrometry. All the additional strains displayed a characteristic [*PSI* ^+^] white and pink coloration when grown on 1/4 YPD plates that was reverted to dark red after prion curing with GdnHCl (Fig. 3a). These strains were picked from a panel of 28 [*PSI* ^+^]_SILAC_ strains. Strains #2 and #3 were generated alongside [*PSI* ^+^]_SILAC_#1, whereas all the other strains were produced in an independent experiment using the original [*psi* ^−^]_SILAC_ cells. The strains were selected to include Sup35 prion with different phenotypic strengths, based on the cell’s ability to grow on plates with media lacking adenine, as well as on media lacking uracil to select for read-through of the *ura3-14* allele that was present on a p*LEU2-ura3-14* plasmid (Fig. 3b, Fig. S4). Strains #1, #2, #15 and #24 displayed more robust growth in comparison to strains #3, #4, #23 and #30.

The eight strains were analyzed by mass spectrometry using SILAC, but without peptide fractionation. Approximately 1,000 proteins (≥2 peptides) were quantified from each sample. When we plotted the log_2_ ratios (H/L) of proteins with respect to their chromosomal localization, it became apparent that two additional strains were also aneuploidic: similar to strain #1, strain #24 was disomic for chromosome I, and strain #30 was disomic for chromosome V (Fig. 3c). Chromosome I disomy was also found in a third [*PSI* ^+^] strain derived from *ltn1∆* cells for an independent study (data not shown). These results indicate that there was likely a higher occurrence of aneuploidy in selected [*PSI* ^+^] strains, especially with a disomy in chromosome I. The acquisition of disomy was first surprising, as aneuploidy was reported to reduce fitness in yeast cells^25^. However, chromosome I is the shortest yeast chromosome and a second copy was not found to significantly reduce cell growth in the W303 background, while disomy of chromosome V was found to slightly reduce cell growth rate^25^.

### [*PSI*^+^] does not induce increased chromosomal instability

**Figure 4:**
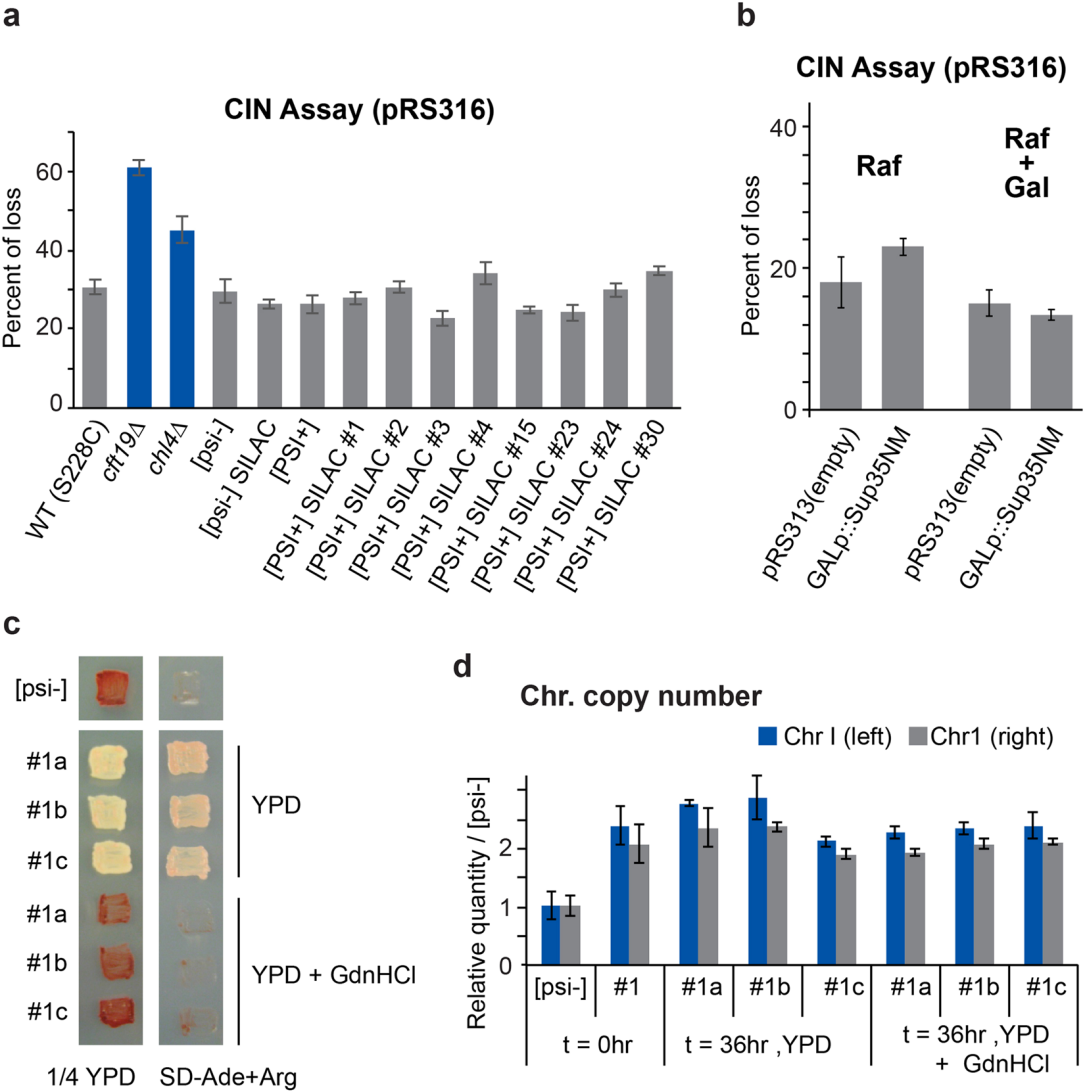
Chromosome instability (CIN). (**a**) The indicated strains transformed with pRS316 were plated on YPD to allow the formation of colonies for 2 days. The colonies were then replicated on SD+Arg−Ura plates. The percentage loss of the *URA3*-containing plasmid was calculated by comparing the number of colonies on each plate: (YPD − SD)/YPD. The standard deviations were calculated from 5 replicates. (**b**) The percentage of pRS316 loss was calculated as in a, but after the incubation of [*psi* ^−^]_SILAC_ cells transformed with the indicated *HIS3* plasmids in YP-raffinose media with or without galactose for 48 hours. (**c**,**d**) Relative quantity of DNA from chromosome I left and right arm compared to [*psi* ^−^], using qPCR. Genomic DNA was extracted from [*PSI* ^+^] #1 samples grown in YPD before (t = 0hr; YPD) and after curing in 4 mM guanidine hydrochloride (GdnHCl) (t = 36 hr; YPD + GdnHCl). [*psi* ^−^] was used as a control, as well as [*PSI* ^+^]#1 grown for 36 hrs in YPD alone (t = 36 hr; YPD). Strains were verified to be cured by plating on 1/4 YPD (no GdnHCl) and SD−Ade+Arg (**c**). Gene regions from chromosome 1 left and right arms were done in triplicates, while error bars represent standard deviation (**d**).

We first reasoned that [*PSI* ^+^] could induce chromosomal instability (CIN). Thermosensitive mutations of both *SUP35* and *SUP45* were proposed to induce CIN^26^, and we reasoned that compromised Sup35 in [*PSI* ^+^] cells could also lead to a similar phenotype. We performed a first series of CIN experiments by monitoring the loss of a *CEN*-containing plasmid. Cells were transformed with pRS316 (*URA3*) plasmid, grown on YPD plates and then replicated on SD−Ura+Arg plates to determine the number of colonies that lost the plasmid. In this assay, there was a basal loss of the *URA3* plasmid in ~30% of the wild type colonies, that was increased to ~45 and 60% in *ctf19Δ* and *chl4Δ* cells, respectively (Fig. 4a), which were previously found to have higher CIN^27^. In contrast, none of the assessed [*PSI* ^+^] strains displayed any major increase of CIN, while there was a minor increase in plasmid loss in #4 and #30 (Fig. 4a). We obtained similar data when we assessed the other 20 [*PSI* ^+^]_SILAC_ strains (data not shown). We also did not observe an increased CIN using a bimater assay that assayed the loss of chromosome III in diploids cells derived from some of the [*PSI* ^+^]_SILAC_ strains analyzed by mass spectrometry (data not shown). We concluded that presence of [*PSI* ^+^] itself did not induce CIN.

We next hypothesized that the CIN could be induced during the conversion of [*psi* ^−^] to [*PSI* ^+^], when the Sup35 fragment is overexpressed. Therefore, we monitored the loss of a *LEU2*-containing plasmid after incubating the [*psi* ^−^]_SILAC_ cells for 48 hours in media with raffinose alone (i.e., no overexpression of the Sup35 fragment) or with galactose. We observed similar levels of plasmid loss when the Sup35NM was overexpressed with galactose in comparison to cells carrying out the control pRS313 plasmid (Fig. 4b). Similar results were obtained when the full length Sup35 was overexpressed from the *CUP1* promoter (Fig. S5a). It should be noted that in contrast to the aforementioned experiments with the *LEU2*-containing plasmid, there was an increase in loss of the *HIS3*-containing plasmid that contained *GALp::sup35NM* upon *GAL* induction, presumably due to negative selective pressure caused by the toxicity of the Sup35NM overexpression (Fig. S5b). Nevertheless, we concluded that the conditions used to induced [*PSI* ^+^] were likely not causing abnormal levels of CIN that could explain the higher occurrence of disomic cells among [*PSI* ^+^] cells.

### Selective pressure and aneuploidy in [*PSI*^+^] cells

We next hypothesized that the recurrent presence of an additional chromosome among [*PSI* ^+^] cells could be due to a possible selective advantage in our growth conditions. We first verified that the chromosome I disomy in our cells was not purely dependent on [*PSI* ^+^]. We cured the prion by growing the [*PSI* ^+^]_SILAC_ #1 cells in biological triplicates (a to c) for 36 hours in YPD supplemented with 4 mM GdnHCl transiently, followed by growth in YPD non-selective media to harvest cells, and then confirmed that the additional chromosome I was still present (Fig. 4c and d). These results also indicate that presence of an additional chromosome I was neither deleterious nor strongly selected against.

**Figure 5:**
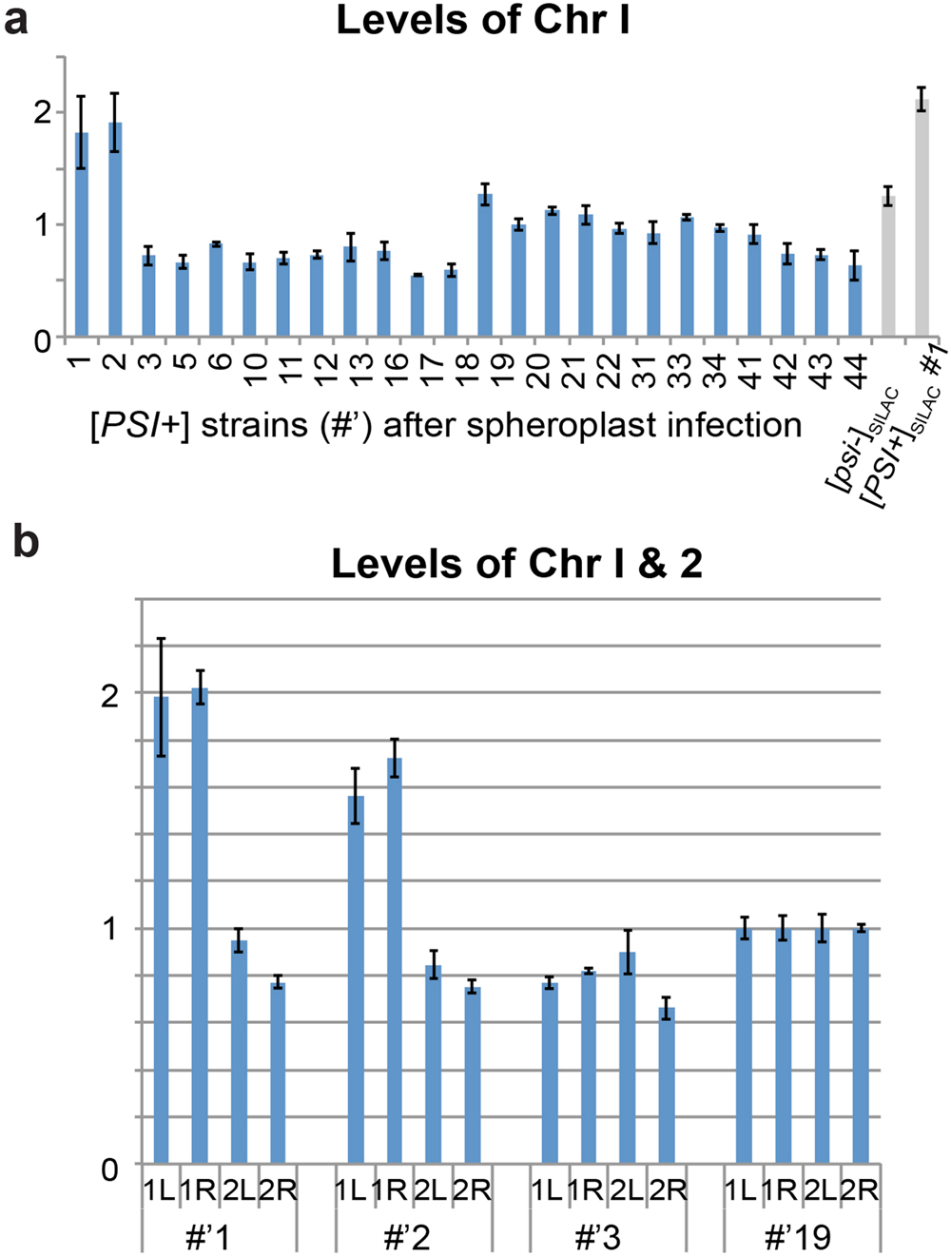
Disomy upon protein-based [*PSI* ^+^] induction. (**a**) 24 strains verified to be [*PSI*+] by colouration were assessed for chromosome 1(Chr I) disomy by qPCR using primers against Chr I left arm. Relative Chr I copy number was determined compared to strain #’20. #’ designates strains that were generated by infecting spheroplasts with prion-containing yeast extract. (**b**) Candidate aneuploid strains #’1 and #’2, along with strains #’3 and #’19, were assessed for Chr I copy number by qPCR with Chr II as comparison, for both left and right chromosomal arms. Chromosome copy number was determined relative to strain 19. Error bars show standard deviation for technical triplicates.

As the previous experiments we used to generate [*PSI* ^+^] strains were carried out by overexpression of the Sup35NM domain, we therefore wanted to determine whether alternate prion induction methods would also yield increased frequency of chromosome I disomy. A new set of [*PSI* ^+^] strains was produced by polyethylene glycol (PEG)-mediated transformation of [*PSI* ^+^] prion-containing yeast extract into S288C [*psi* ^−^] spheroplasts, adapted from previously published protocols^28–30^. Strains were selected by growth on SD−Ade+Arg plates after 10 days and confirmed to be [*PSI* ^+^] by GdnHCl reversibility of coloration (Fig. S6a; these strains were thereafter denoted #’). Of 24 newly generated strains verified to be [*PSI* ^+^] that were analyzed for chromosome I disomy by qPCR, we found that two strains (#’1 and #’2) had acquired an additional chromosome I (Fig. 5a and b). These were the first two colonies to originally appear on the SD−Ade+Arg selection plate and were the largest and whitest colonies present. Comparison of colony growth on SD−Ade+Arg plates indicate that these two chromosome I disomic strains indeed result in larger colonies than strains #’3 and #’19 that were generated at the same time, while no difference in growth was seen on YPD non-selective media (Fig. S6b,c). This strongly suggests that the presence of an additional chromosome I may provide a growth advantage on SD−Ade+Arg media for cells with the *ade1-14* mutation, possibly via a compensatory mechanism.

**Figure 6:**
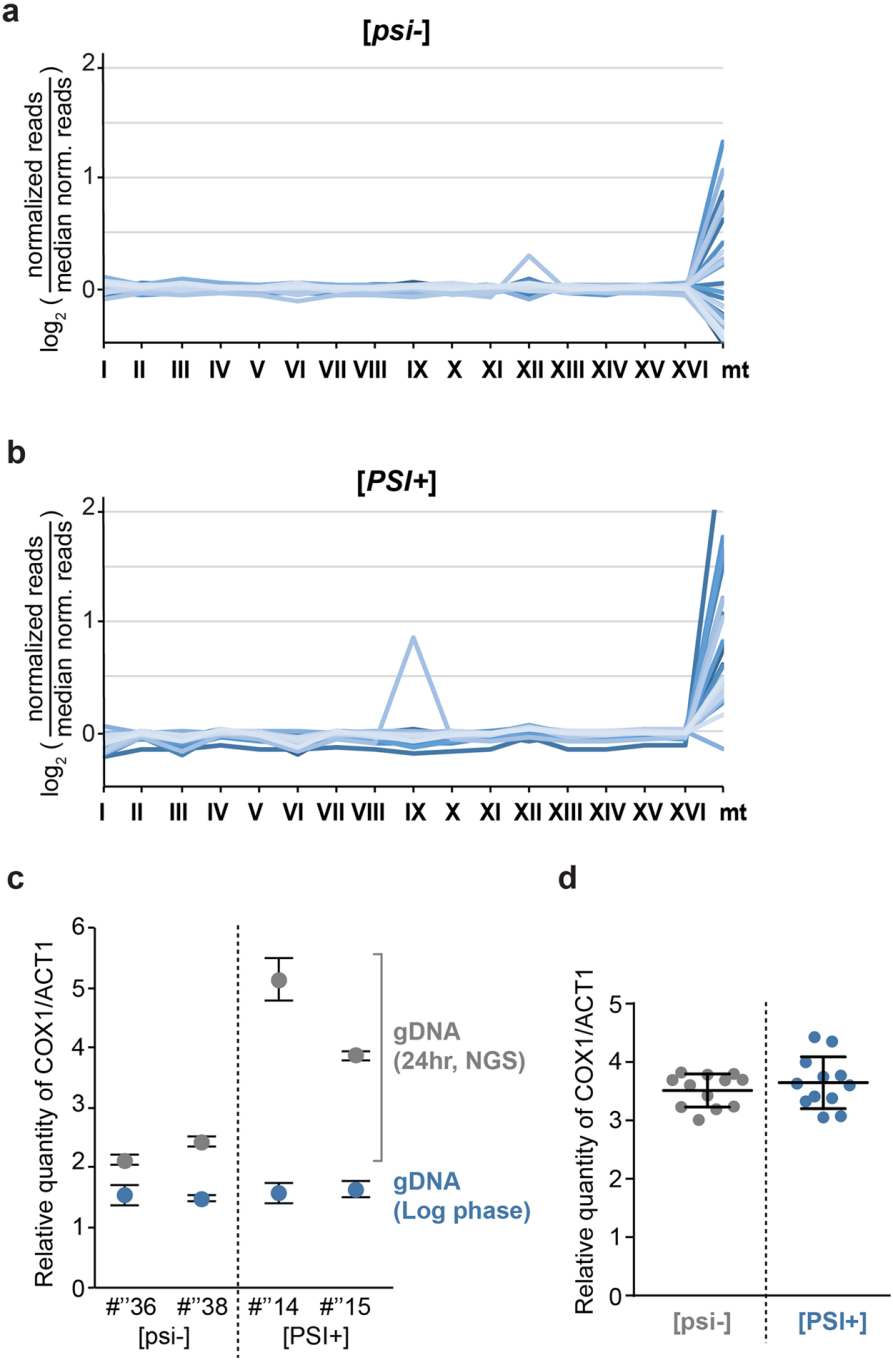
Survey of chromosome copy number by whole genome sequencing. (**a**,**b**) For sequencing reads matching each indicated chromosome, the log_2_ ratios of the “normalized reads” (*i*.*e*., percent of reads matching a given chromosome in a given strain) divided by the “median normalized reads” (*i*.*e*., median of percentages of reads matching a given chromosome in all [*psi* ^−^] strains) were reported for 30 [*psi* ^−^]_SILAC_ strains (**a**) and 30 [*PSI* ^+^]_SILAC_ strains (**b**) selected on YPD plates after Sup35NM overexpression. Each strain is represented by a line in a different blue colour. (**c**) mtDNA levels of the indicated strains were quantified by comparing relative mitochondrial *COX1* vs. nuclear *ACT1* genes using qPCR. The genomic DNA (gDNA) used for the NGS analysis was analyzed (grey), as well as gDNA obtained from the same strains that were kept in the logarithmic growth phase (blue). Standard deviations are from three technical replicates. #” designates strains that were generated for the whole genome analysis. (**d**) 12 [*psi* ^−^] and [*PSI* ^+^] strains previously used for NGS were grown for 24 hours in YPD with excess adenine (100 mg/mL) to stationary phase. mtDNA levels were then quantified by comparing relative mitochondrial *COX1* vs. nuclear *ACT1* genes using qPCR.

The *ade1-14* allele, which is used for [*PSI* ^+^] selection, is actually located on chromosome I. The presence of an additional chromosome I could therefore constitute a selective advantage in absence of adenine, as increased *ade1-14* mRNA would lead to more Ade1. To test this idea, we generated a novel set of [*PSI* ^+^] strains but without the selection pressure caused by the lack of adenine. We posit that chromosome I disomy would no longer be prevalent in this case. After inducing Sup35NM expression with galactose, the cells were plated on YPD media, on which both light pink [*PSI* ^+^] and red [*psi* ^−^] grew. In contrast, [*psi* ^−^] cells did not grow in our previous conditions when using SD−Ade+Arg plates. We selected 30 red colonies that remained [*psi* ^−^] and 30 light pink colonies that were [*PSI* ^+^], based on the reversibility of the phenotype in GdnHCl (Fig. S7a), and contain prions of various strengths (Fig. S7b). These strains were analyzed by next generation sequencing (NGS) to assess their copy number of chromosomes based on sequencing reads. The average reads per chromosomes was compared for each strain. Strikingly, no chromosome I disomy among all strains was observed, while one [*PSI* ^+^] strain was disomic for chromosome IX (Fig. 6a and b). These results indicate that in absence of positive selection pressure, there was no particular increase of chromosome I disomy among [*PSI* ^+^] strains.

Interestingly, we observed that the number of sequencing reads for the mitochondrial chromosome (mtDNA) was generally higher in [*PSI* ^+^] strains (Fig. 6a and b), and that this difference was statistically significant (Fig. S8a). One concern is that as the number of mtDNA copy increases after the diauxic shift^31^, the higher number of sequencing reads for mtDNA in [*PSI* ^+^] could be caused by a longer growth phase due to increased read-through of *ade1-14* in media with lower amounts of adenine. For instance, [*PSI* ^+^] cell cultures reached a higher O.D._600_ in comparison to [*psi* ^−^] cells when grown to saturation in standard YPD media, but not when adenine was added to an excess (Fig. S8b). For selected strains, we confirmed by qPCR that mtDNA levels were lower in both [*psi* ^−^] and [*PSI* ^+^] logarithmic growing cells, in comparison to levels obtained when preparing DNA for the NGS analysis (in the latter case, cells were grown for 24 hours including an extended period post the diauxic shift in which cells rely on aerobic respiration) (Fig. 6c). Two of the [*PSI* ^+^] selected strains (#”14 and #”15) contained a high percentage sequencing counts matching mtDNA (10.3 and 4.5%), whereas the two [*psi* ^−^] strains (#”36 and #”38) had lower percentages (1.6 and 1.9%) in the NGS analysis. Importantly, the mtDNA levels were indistinguishable between the [*PSI* ^+^] and [*psi* ^−^] cells in logarithmically growing cells (Fig. 6c). As well, we found that there was no statistical difference in mtDNA levels between [*psi* ^−^] and [*PSI* ^+^] cells when cells were grown for 24 hours until saturation in YPD in presence of excess amount of adenine (Fig. 6d; p = 0.53). However, we noted that there was a higher variability in mtDNA copy number in [*PSI* ^+^]. These results indicate that [*PSI* ^+^] was not directly leading to higher levels of mtDNA when growth conditions were carefully established to take in account the presence of the *ade1-14* allele. In agreement, there appears to be no distinct correlation between mtDNA levels and [*PSI* ^+^] prion strength (data not shown).

It was previously indicated that [*PSI* ^+^] caused mitochondria fragmentation and a reduction of several mitochondrial proteins based on mass spectrometry analysis of purified mitochondria^32^. However, we did not observe lower levels of mitochondrial proteins (encoded from both mitochondrial or nuclei genome) in our mass spectrometry analysis of the supernatant fraction (which contains these proteins). We also found that selected [*PSI* ^+^] strains grew normally in comparison to [*psi* ^−^] cells with media containing glycerol, a non-fermentable carbon source that imposes respiratory growth (Fig. S8d). These results indicate that mitochondria function was not impaired in the [*PSI* ^+^] strains generated in this study.

## Discussion

In this study we compared the proteome composition of cells with or without the [*PSI* ^+^] prion. Strikingly, no major change was observed under standard growth conditions. A recent study showed that a number of genes were repressed in [*PSI* ^+^] cells compared to [*psi* ^−^] based on transcriptome analysis^16^. However, we were unable to confirm that these proteins were expressed at lower levels in our experimental set up, with the exception of Gre2 and YNL134C. This may be due to changes in protein stability, variations in the genetic background, strength of the [*PSI* ^+^], or differences in laboratory growth conditions. Interestingly though, we found that several [*PSI* ^+^] strains we selected contained an extra chromosome I in [*PSI* ^+^]. We attributed this phenomenon to the selective pressure that we applied during the selection of [*PSI* ^+^] cells.

As [*PSI* ^+^] cells display a higher rate of translational read-through events, the presence of extended C-terminal domains could: 1) impart an alternative function, 2) increase stability, or 3) decrease stability in comparison to the native form. Despite a good coverage of the proteome (>4000 proteins), we only identified two peptides to be C-terminal extended peptides with good confidence. Although we also attempted to employ C-terminal amine-based isotope labeling of substrates (C-TAILS) approach to enrich for C-terminal peptides^33, 34^, the low coverage of the current method prevented identification of C-terminal peptides that were more enriched in [*PSI* ^+^] cells (data not shown). One possibility is that these C-terminal extended proteins are present in much lower abundance in the cells in comparison to proteins that were not extended. In yeast and other eukaryotes, C-terminally extended proteins have the potential to greatly alter the original protein’s intended function by altering protein conformation and stability^35^. In order to mitigate the occurrence of undesired read-through events, *S*. *cerevisiae* has evolved to harbor a higher frequency of in-frame stop codons especially within the first few nucleotides downstream of the original stop codon^36^. This acts redundantly as a safety feature to prevent the production of elongated C-terminal extended proteins with aberrant and potentially deleterious functions. Notably, for some extended polypeptides that have bypassed additional stop codons downstream of the open reading frame (ORF), they may be directly degraded by the Ltn1 quality control E3 ligase if the poly(A) tract is also translated^37^. Another possibility is that sufficient levels of Sup35 remain functional in the S288C [*PSI* ^+^] variant we examined, thereby greatly reducing the rate of read-through events. Serio and colleagues indeed found that Sup35 amyloid-aggregates were not only capable of interacting with eRF1 and ribosomes to retain translation termination function, but that this ability greatly depended on physical characteristics of the aggregates and contributed to prion strain phenotype^11^. Furthermore, genetic background of our S288C variants may also play a key role in the extent of translation read-through. Different strains such as the commonly used 74-D694 have thousands of single-nucleotide polymorphisms (SNPs) compared to S288C, which may encode additional inactivating stop codon mutations^38^. Genetic background has been indeed noted to intricately influence phenotype in [*PSI* ^+^] strains^13, 39^. It is worth mentioning that in contrast, other strains may also display abnormally enhanced read-through due to the reduced translation fidelity that can be caused for instance by mutations in tRNA, such as SUQ5/SUP16^24^; therefore, caution should also be applied when using these strains.

Among the 9 [*PSI* ^+^] strains that we assessed and were generated in three separate experiments by galactose-induced Sup35NM overexpression (that include the 8 strains generated in two independent experiments presented in this study), three strains were disomic for chromosome I and one for chromosome V. Whereas a *sup35* mutant was reported to induce CIN^26^, we found no evidence that [*PSI* ^+^] induces CIN. Chromosome I is the shortest in *S*. *cerevisiae* and previous work showed that chromosome I disomy did not reduce cell fitness in a noticeable manner^25^. We posit that the presence of *ade1-14* on chromosome I is likely the main driver for the retention of an additional copy of that chromosome to increase levels of Ade1 when cells are grown in absence of adenine. When we utilized a different approach to generate [*PSI* ^+^] strains via transformation with prion-containing yeast extract, two strains out of 24 carried out an extra chromosome I. These two strains grew faster and larger than other colonies on the SD−Ade+Arg selection plate, indicating a possible growth advantage. In further support, we found no chromosome I disomy among the 30 [*PSI* ^+^] strains that we generated in non-selective conditions with the presence of adenine in plate media. These results indicate that selective pressure applied during the generation of prion cells may have unsought consequences. It is important to note however that due to the scope of this study, we only assessed S288C cells; thus it is possible that the frequency of aneuploidy upon adenine selection pressure may differ between strains, as genetic background has been found to affect the rate of chromosomal rearrangements in yeast^40^.

One question that still remains is how selection may favour the isolation of these aneuploidic [*PSI* ^+^] strains. At first glance, the odds of survival seem stacked against these cells that already harbor Sup35 amyloid-aggregates, as aneuploidy in yeast has been linked with proteotoxic stress and increased aggregation formation, and are generally found to have lower fitness under normal conditions^41, 42^. However, aneuploidy may potentially confer a growth advantage given certain circumstances in which an additional chromosome acts in a compensatory manner, such as in deletion mutants by increasing dosage of a gene encoding a protein with similar function to the deleted one^43^, or by introducing phenotypic traits that allow for increased fitness in stressful environments^42^. We speculate that in our case during prion induction, random errors during mitosis may have resulted in a small population of [*PSI* ^+^] aneuploids forming that in non-selective conditions otherwise may potentially die out, but if disomic for chromosome I, are able to grow and establish first on the SD−Ade+Arg media. For newly generated [*PSI* ^+^] strains establishing on adenine-deficient media, the extra chromosome I copy may have allowed for twice as much Ade1 to be synthesized from *ade1-14* read-through. This could result in faster colony growth, and thereby selection of chromosome I disomic strains.

During the NGS analysis we also observed higher average levels of mitochondrial DNA among [*PSI* ^+^] strains. We believe this was due to a possible prolonged growth of [*PSI* ^+^] cells after the diauxic shift when mitochondrial DNA is duplicated due to a better ability of the prion cells to synthesize adenine in presence of the *ade1-14* allele. These results highlight how specific experimental conditions may introduce phenotypic traits in [*PSI* ^+^] cells that are linked to the markers introduced to monitor the presence of the prion protein (e.g., *ade1-14*). For instance, this may explain why [*PSI* ^+^] cells typically displayed a distinct slower growth recovery in comparison to [*psi* ^−^] cells after an oxidative stress, when cells were grown to saturation (data not shown). Altogether our work suggests that the presence of [*PSI* ^+^] does not induce a major conversion of the proteome composition in standard growth conditions and that selective pressure in presence of auxotrophic markers with nonsense mutations may introduce undesired effects. For future studies, it will be important to determine whether [*PSI* ^+^] may induce greater changes in the proteome composition in specific stress conditions or conditions that affect translation fidelity, especially given the significant induction of the prion under such conditions^44^.

## Methods

### Generation of auxotrophic [*psi*^−^]_SILAC_ and [*PSI*^+^]_SILAC_ strains

*Saccharomyces cerevisiae* strains (S288C background) are listed in Supplementary Table 1. The original *ade1-14* containing cells that were [*PIN* ^+^] and either [*psi* ^−^] or [*PSI* ^+^] strains were generated by transformation of S288C with a PCR product carrying the *ade1-14* reporter gene linked to the nourseothricin-resistance cassette (NAT), followed by selection on YPD + nourseothricin. The [*PSI* ^+^] prion was induced in the resulting strain as described below. Successful induction of the prion confirmed the [*PIN* ^+^] status of the strains. The [*psi* ^−^]_SILAC_ strains were first generated by mating and sporulating the S288C [*psi* ^−^] with our SILAC strain (*lys2∆*, *arg4∆*) (YTM1173) and all [*PSI* ^+^]_SILAC_ strains were induced from the same [*psi* ^−^]_SILAC_ strain. For all but one experiment (see next paragraph), the induction of [*PSI* ^+^] was performed as previously published^45^. In brief, [*psi* ^−^]_SILAC_ were first transformed with the pRS313 plasmid that contains *GALp::Sup35NM-GFP* ^46^ (BPM915) that expresses the first 253 amino acid of Sup35 and can induce the conversion of [*psi* ^−^] to [*PSI* ^+^]. Cells were grown in SD−His+Arg in presence of 2% raffinose at 30 °C for 2 days and then diluted to an O.D._600_ of 0.1 in SD−His+Arg in presence of 2% galactose for 2 days. About 100 cells were then plated on SD−Ade−His+Arg (2% dextrose) and white yeast colonies were selected after 5 days at 25 °C. In these conditions, about 1–2% cells grew on the selective plate in comparison to cells grown on YPD plates. Alternatively, cells were only plated on YPD and candidate [*psi* ^−^] or [*PSI* ^+^] colonies were selected based on coloration (red or white, respectively).

[*PSI* ^+^] induction by protein transformation with crude yeast extract containing [*PSI* ^+^] prions was performed as previously conducted, with some modifications^29, 30^. Briefly, logarithmically growing [*psi* ^−^]_SILAC_ and [*PSI* ^+^]_SILAC_ #15 cells were harvested at 1 O.D._600_ (optical density at 600 nm) and lysed with glass beads in 50 mM Tris-HCl (pH 7.5), 200 mM NaCl, 2 mM TCEP, 5% glycerol, 1 mM EDTA, and 1x PIC (protease inhibitor cocktail, Roche). The Precellys®24 (Bertin Corp) was used for glass bead lysis at 5000 rpm for 2 × 20 sec at 4 °C. Cell lysates were precleared at 500 g for 2 min at 4 °C and the *DC* Protein assay (Bio-Rad) was used to measure total protein concentration of the samples. [*psi* ^−^]_SILAC_ spheroplasts were then produced by collecting 50 mL logarithmically growing YPD culture grown to ~0.5 O.D._600_ at 30 °C by centrifugation at 3,000 g for 5 min. Cells were washed successively using 20 mL water; 1 M sorbitol; followed by SCE buffer (1 M sorbitol, 10 mM EDTA, 10 mM DTT, 100 mM sodium citrate, pH 5.8) and centrifuged at 3,000 g for 5 min. Cells were then resuspended in 1 mL SCE and incubated with 200 units/mL lyticase (Sigma-L2524) for 30 min at 30 °C to generate spheroplasts. Further centrifugations were done at 400 g for 5 min. Spheroplasts were washed with 20 mL 1 M sorbitol, followed by 20 mL STC buffer (1 M sorbitol, 10 mM CaCl_2_, 10 mM Tris-HCl, pH 7.5), and resuspension in 1 mL STC. 25 µL of the above lysis buffer or 2 mg/mL [*psi* ^−^]_SILAC_ or [*PSI* ^+^]_SILAC_ #15 crude cell lysate were mixed with 10 µL ssDNA and added to 100 µL prepared spheroplasts, then incubated for 30 min at 25 °C. Fusion was induced by adding 9 volumes of PEG-buffer (20% w/v PEG 8000, 10 mM CaCl_2_, 10 mM Tris-HCl, pH 7.5) and incubating for 30 min at 25 °C. Spheroplasts were collected at 400 g for 5 min and resuspended in 150 µL SOS buffer (1 M sorbitol, 0.25% yeast extract, 0.5% bacto-peptone, 7 mM CaCl_2_), then incubated for 30 min at 30 °C. After, spheroplasts were mixed with 7 mL SD−Ade+Arg top agar (2.5% agar with 1 M sorbitol and 2% dextrose, heated to 45 °C) and poured onto SD−Ade+Arg plates containing 1 M sorbitol and 2% dextrose. Plates were incubated at 30 °C for 10 days, with colonies only being observed for [*psi* ^−^]_SILAC_ spheroplasts transformed with [*PSI* ^+^]_SILAC_ #15 lysate.

To validate presence of [*PSI* ^+^], strains were grown to stationary phase overnight at 30 °C and then spotted at 1 O.D._600_ on SD−His+Arg, 1/4YPD, and 1/4YPD + 4 mM GdnHCl plates and grown for 3 days at 30 °C. For assessing prion strength, all strains with pLEU2-*ura3-14* plasmid^46^ (BPM916) were grown to stationary phase in SD−Leu+Arg overnight at 30 °C. Cells were spotted in 10-fold serial dilutions onto SD−Leu+Arg, SD−Leu-Ura+Arg and SD − Ade + Arg, starting at 1 O.D._600_, and then incubated at 30 °C.

In order to assess [*PSI* ^+^] stability of our strains during thermal stress based on a previously published assay^22^, indicated strains were grown overnight at 30 °C and while in log phase, were plated on 1/4 YPD (low adenine content) to best observe any colour differences that may occur. Strains were grown for ~5 hrs until approximately 8 cells were observed per colony, after which plates were incubated at either 30 °C or 40 °C for 30 min. Cells were then re-incubated at 30 °C for several days and assessed for colony sectoring.

### SDD-AGE

Semi-denaturing detergent agarose gel electrophoresis (SDD-AGE) was performed based on previously published protocol^47^. Cells were resuspended in PEB buffer (25 mM Tris-HCl, 100 mM NaCl, 10 mM MgCl_2_, 1 mM EDTA, 1 mM DTT, 2 × PIC, pH 7.5) and lysed by glass beating with the Precellys®24 device (Bertin Corp) at 4 °C. The lysates were precleared by centrifugation at 300 g at 4 °C for 15 min and supernatant was collected. 100 µg of proteins in 4 × Sample Buffer (2 × TAE, 20% glycerol, 8% SDS, 0.03% (w/v) bromophenol blue) were loaded into 1.5% agarose gel in 1 × TAE (40 mM Tris, 20 mM acetic acid, 1 mM EDTA, pH 7.6) containing 0.1% SDS. The gel was run at 120 V at 4 °C until the dye front reached 1 cm from the end of the gel. The proteins were transferred onto nitrocellulose membrane at 25 V at 4 °C for 16 hours. Sup35 monomers and amyloids were subsequently immuno-detected using anti-Sup35 polyclonal antibody (Santa Cruz, sc-25915, 1:1,000 dilution) and the IRDye® 800CW Donkey anti-Goat secondary antibody (LI-COR, 925-32214, 1:20,000 dilution) by standard Western blot and by imaging with the LI-COR Odyssey system.

### Preparation of cell lysates

[*psi* ^−^]_SILAC_ and [*PSI* ^+^]_SILAC_ cells (three biological replicates) were grown in YNB media (ThermoFisher Scientific) containing 0.5% (w/v) dextrose and 0.002% (w/v) adenine, tryptophan, methionine, uracil and leucine and supplemented with either light amino acid (Lys_0_, Arg_0_) for [*PSI* ^+^]_SILAC_ or heavy amino acid (Lys_4_, Arg_6_) for [*psi* ^−^]_SILAC_ (Cambridge Isotopes) (30 mg/L for Lys and 20 mg/L for Arg). The cells at log phase were harvested by centrifugation at 4,000 rpm for 5 minutes at 4 °C and were washed twice with ice-cold TBS (50 mM Tris-HCl, 150 mM NaCl, pH 7.5). Light and heavy labelled cells were combined in 1:1 ratio according to the O.D._600_. For total crude cell lysate samples, a portion of the combined cells were collected separately, flash-frozen in N_2(*l*)_, and stored at −80 °C before further processing. The rest of the cells were resuspended in the same volume of 2 × Lysis Buffer (200 mM HEPES, 600 mM NaCl, 2 mM PMSF, 2 × PIC, pH 7.5) and added drop-by-drop into N_2(*l*)_ to form “cell beads” that were then stored at −80 °C.

For total crude lysate sample, 200 µL of HU-SDS buffer (100 mM HEPES, 8 M urea, 0.1% SDS, pH 8.0) was used to resuspend the frozen cells. Cells were lysed by glass bead beating with the Precellys®24 device (Bertin Corp) at 4 °C. Cell lysates were collected in a clean tube. Additional 200 µL of HU buffer (100 mM HEPES, 8 M urea, pH 8.0) was used to rinse the glass beads and combined with the sample. The collected cell lysates were mixed by pipetting up and down and centrifuged at 16,000 × g at 4 °C for 15 min. The supernatant containing the cleared crude cell lysates was collected for further processing.

Soluble and pellet fractions were generated as before^48^. Frozen “cell beads” were placed onto a N_2(*l*)_ pre-cold mortar and lysed by cryo-grinding. After thawing lysates on ice, Triton X-100 was gently added to the cell lysates to a final concentration of 1%. The cell lysates were first precleared by centrifuging at 300 g at 4 °C for 15 min, and then centrifuged at 16,000 g at 4 °C for 15 min to separate the soluble and “sedimentable” (pellet) fractions. The supernatants were collected and the pellets were washed twice with Lysis Buffer (100 mM HEPES, 300 mM NaCl, 1 mM PMSF, 1 × PIC, pH 7.5), and then resuspended in HU-SDS buffer and vortexed at room temperature for one hour. The mixture was centrifuged at 16,000 g for 15 min and supernatant containing dissolved proteins was collected.

Total crude lysate and soluble samples were subjected to a methanol-chloroform protein precipitation^49^ to remove detergent and were resuspended in HU buffer. Pellet samples were subjected to a Detergent Removal Spin Column (Pierce; ThermoFisher Scientific). Protein concentrations were determined by the DC Protein Assay (BioRad).

### Preparation of mass spectrometric samples and offline fractionation

The samples were reduced using 5 mM TCEP for 20 min, and alkylated by 50 mM chloroacetamide for 30 min in the dark at room temperature. The proteins were first digested with endo-proteinase LysC (Roche, 11047825001, 1:100 w/w) at 35 °C for an hour. The mixture was then diluted to 2 M urea with tryptic digestion buffer (100 mM Tris-HCl, 1 mM CaCl_2_, pH 8.5) and the proteins were further digested by trypsin (Thermo, PRV5113, 1:20 w/w) for 20 hours at 35 °C. The resulting peptides from total crude lysate and soluble fractions were purified by STop-And-Go Extraction (STAGE) high-capacity tips using C18 resin (Phenomenex)^50^.

Roughly 100 μg of peptides of each sample was fractionated by offline high pH reversed-phase chromatography using a Zorbax Extend-C18 analytical column, 5 μm, 4.6 × 50 mm (Agilent), on a 64 minute gradient (followed by a 21 minute equilibration with buffer A) with a 50 μL/min flow rate, where Buffer A contained 5 mM NH_4_HCO_2_, pH 10 and 2% acetonitrile and Buffer B contained 5 mM NH_4_HCO_2_, pH 10 and 90% acetonitrile. Ninety-six fractions were collected at 40 seconds/fraction. The resulting fractions were pooled in a non-contiguous manner, as previously described^51^. The fractionated samples were pooled into 9 fractions.

### Mass spectroscopy, data processing and analysis

Purified peptides were resuspended in Buffer A (see below) and analyzed using an orthogonal quadrupole – time of flight mass spectrometer (Q-TOF) Impact II (BrukerDaltonics) on-line coupled to an Easy nano LC 1000 nanoflow HPLC (Thermo Scientific) using a Captive Spray nanospray ionization source (BrukerDaltonics) including a 2-cm-long, 100-μm-inner diameter fused silica trap column, 75-μm-inner diameter fused silica analytical column with an integrated 10 μm opening spray tip (pulled on a P-2000 laser puller from Sutter Instruments). The trap column was packed with 5 μm-diameter Aqua C-18 beads (Phenomenex, www.phenomenex.com) while the analytical column was packed with 3 μm-diameter Reprosil-Pur C-18-AQ beads (Dr. Maisch, www.Dr-Maisch.com). Buffer A consisted of 0.1% formic acid in water, and buffer B consisted of 0.1% formic acid in acetonitrile. Gradients were run at 250 nL/min from 10% B to 35% B over 74 min, then the column was washed with 100% B for 15 min. The HPLC auto-sampler was maintained at 7 °C. The Captive Spray Tip holder was modified similarly to an already described procedure^52^ – the fused silica spray capillary was removed (together with the tubing which holds it) to reduce the dead volume, and the analytical column tip was fitted in the Bruker spray tip holder using a 8 mm long piece of 1/16” × 0.015 PEEK tubing (IDEX), an 1/16” metal two way connector and a 16-004 Vespel ferrule. Impact II used OTOF Control 1.8 ver. 4.0.17.1840, HiStar 3.2 and Compass 4.2 (BrukerDaltonic). The Impact II was set to acquire an MS scan for 0.2 sec followed by MS/MS scans for the reminder of a 3 sec period using 0.06 sec per MS/MS scan. The mass range for the MS scan was 300 to 2200 Th and for the MS/MS scans 200 to 2200 Th. The minimum parent ion intensity for triggering MS/MS was 500 counts. Parent ions were then excluded from MS/MS for the next 0.3 min. Singly charged ions and ions with unknown charge were excluded and Strict Active Exclusion was used. Mass accuracy: error of mass measurement is usually within 5 ppm and is not allowed to exceed 10 ppm. The spectra files were processed using MaxQuant (v1.5.3.17)^53–55^. The derived peak list was searched against the reference yeast proteome downloaded from Uniprot (http://www.uniprot.org/) on 04-09-2015 (6674 sequences) and the false discovery rate was set to 1%. For the purpose of generating an extended database of the *Saccharomyces cerevisiae* proteome, the genomic sequences for each open reading frame +1000 nucleotides were downloaded from the SGD database^56^. Using in-house scripts and starting with the genomic database, each of the sequences was translated into the corresponding wild-type protein sequence in addition to three read-through scenarios corresponding to situations where 1, 2 or 3 nucleotides were skipped in case of a +1, −1 or 0 frame shift, respectively, the latter corresponding of the read-though the entire stop codon. In each scenario, the protein sequence was extended until the next stop codon is reached. This resulted in a final database of 22,724 translated sequences in FASTA format. The processed data were analyzed by Perseus (v.1.5.1.6)^57^. Data are available via ProteomeXchange with identifier PXD007168.

### Genomic DNA preparation for qPCR and NGS

Genomic DNA (gDNA) from [*psi* ^−^]_SILAC_ and [*PSI* ^+^]_SILAC_ were prepared from log-phase culture in YPD media, unless specified, whereas the Next-Generation Sequencing (NGS) samples were prepared from stationary-phase culture in YPD media. 5 O.D._600_ of cells were harvested for gDNA extraction using Thermo Scientific GeneJET Genomic DNA Purification Kit (ThermoFisher). gDNA samples were quantified using Qubit® dsDNA HS Assay Kit (ThermoFisher).

### qPCR

The quantitative PCR (qPCR) experiments were performed in 10 µl reactions using the Applied Biosystems ViiA^TM^ 7 Real-Time PCR System (ThermoFisher) following a previously published procedure^42^. Briefly for experiments assessing chromosome I disomy, 4 ng of gDNA and 5 µM of each forward and reverse primer were used in each reaction. The list of primers is provided in Supplementary Table 2. Power SYBR® Green PCR Master Mix (ThermoFisher) was used. The reactions were denatured at 95 °C for 5 minute initially. During the 40 cycles of PCR reaction, denaturation was done at 95 °C for 15 second and extension was performed at 60 °C for 1 minute. For the analysis of mitochondrial gene *COX1* in comparison to nuclear gene *ACT1*, qPCR experiments were performed as above, except that 0.2 ng of gDNA was used and extension was done at 58 °C for 1 min. The primers were from a previously published protocol^58^. The 2^−ΔΔ*CT*^ method^59^ was used to determine relative chromosome or mitochondrial copy number with standard deviations from technical triplicates.

### Chromosome instability (CIN) assay

The yeast strains transformed with pRS316 were plated on YPD to allow the formation of colonies (~100) for 2 days. *ctf19Δ* and *chl4Δ* cells used as CIN positive controls were kindly provided by P. Hieter (University of British Columbia, Canada). The colonies were then replicated on SD+Arg−Ura plates. The percentage loss of the *URA3*-containing plasmid was calculated by comparing the number of colonies on each plate: (YPD-SD)/YPD. The standard deviations were calculated from 5 replicates.

### Next Generation Sequencing (NGS)

Genomic DNA was fragmented to an average size of 250 bp in 40 µL volume of EB buffer (Qiagen) using Covaris E-220 Focused-ultrasonicator (55 sec, 20% duty factor, 200 bursts/min). All liquid handling steps were carried out on the Bravo liquid handling platform using in house created programs with VWorks Automation Control Software (Agilent Automation, Santa Clara, CA, USA). End Repair and 5′ Phosphorylation reaction (40 µL DNA sample, 5.5 µL of 10X NEB 2 buffer, 2.2 µL of 25 mM ATP, 2.2 µL of 10 mM dNTP, 10U T4 Polynucleotide Kinase, 4.5U T4 DNA Polymerase, 1U Klenow Large Fragment DNA Polymerase, and ultrapure water to a total reaction volume of 55 µL (New England Biolabs Ipswich, MA, USA) was incubated for 30 minutes at room temperature. DNA samples were then purified using in house prepared Sera-Mag magnetic bead solution (1 M NaCl, 23% PEG, Sera-Mag Speedbeads (Fisher Scientific, Pittsburgh PA, USA)) with final PEG concentration of 13.8% and eluted in 35 µL volume with Qiagen EB buffer. To enable ligation to the adaptors, a single dA overhang was added to the 3′ ends of DNA fragments (35 µL DNA, 5 µL of 10X NEB 2 buffer, 1 µL of 10 mM dATP, 5U Klenow Fragment (3′ → 5′ exo–), and ultrapure water to a total reaction volume of 50 µL, (New England Biolabs Ipswich, MA, USA)). The reaction was incubated at 37 °C for 30 minutes. The product was then purified using in house-made Sera-Mag magnetic bead solution with final PEG concentration of 13.8% and eluted in 35 µL volume with Qiagen EB buffer. Next, short adaptors containing sequences required downstream in the sequencing workflow were ligated to the dA-tailed DNA fragments (35 µL DNA, 12 µL of 5X Quick Ligation Buffer, 2000U Quick T4 DNA Ligase, 2 µL of 0.5 µM Illumina short sequencing adaptor, and ultrapure water to a total volume of 60 µL). Ligation reaction was performed at room temperature overnight. The ligation product was then purified using in house-made Sera-Mag magnetic bead solution with final PEG concentrations of 10.2% and eluted from the beads with 35 µL of Qiagen EB buffer.

Adaptor ligated libraries were PCR amplified and barcoded by custom indexing primers in a 60 µl PCR reaction (35 µL DNA, 12 µL of 5X High fidelity buffer, 1 µL of 10 mM dNTPs, 1.5 µL of DMSO, 1 µL of 25 µM PCR primer 1.0, 2 µL of 12.5 µM custom indexing primer (added separately to each well), 1U Phusion Hot Start II, and ultrapure water to a total volume of 60 µL (Fisher Scientific, Pittsburgh PA)). PCR amplification was carried out with the following cycling conditions: 98°C for 30 seconds, 6 cycles of (15 seconds at 98 °C (denaturation), 30 seconds at 65 °C (annealing), 30 seconds 72 °C (extension)), followed by 5 min at 72 °C. PCR amplified libraries were cleaned up with in house-made Sera Mag magnetic bead solution with final PEG concentration of 10.5% and eluted with 35 µL of Qiagen EB buffer. Libraries were quantified using Qubit HS DNA assay, pooled equal-molar for MiSeq sequencing. 3.7GB of data was generated in a PE 75 bp run with base quality of 97% of Q30 or above. Adapter trimming and alignment to sacCer3 genome was performed on the MiSeq with alignment rates ranging between 95–99%. Samtools V0.1.19 (https://www.ncbi.nlm.nih.gov/pubmed/19505943) was used to extract the number of reads per chromosome per sample. These counts were then normalized to the library depth by dividing by the total number of aligned reads per sample.

## Electronic supplementary material

Supplementary Information

Supplementary Data
