# Supplementary material for: The [PSI+] yeast prion does not wildly affect proteome composition whereas selective pressure exerted on [PSI+] cells can promote aneuploidy
Source: Sci Rep. 2017 Aug 16;7:8442. doi: 10.1038/s41598-017-07999-8 (PMC5559586; doi:10.1038/s41598-017-07999-8)
Supplement: Supplementary file 1 — Supplementary Information [file 41598_2017_7999_MOESM1_ESM.pdf]

Supplementary Information

The [*PSI*$^+$] yeast prion does not wildly affect proteome composition whereas selective pressure exerted on [*PSI*$^+$] cells can promote aneuploidy.

Patrick H. W. Chan*, Lisa Lee*, Erin Kim, Tony Hui, Nikolay Stoynov, Roy Nassar, Michelle Moksa, Dale M. Cameron, Martin Hirst, Joerg Gsponer, Thibault Mayor

* equal contributions

Figure S1

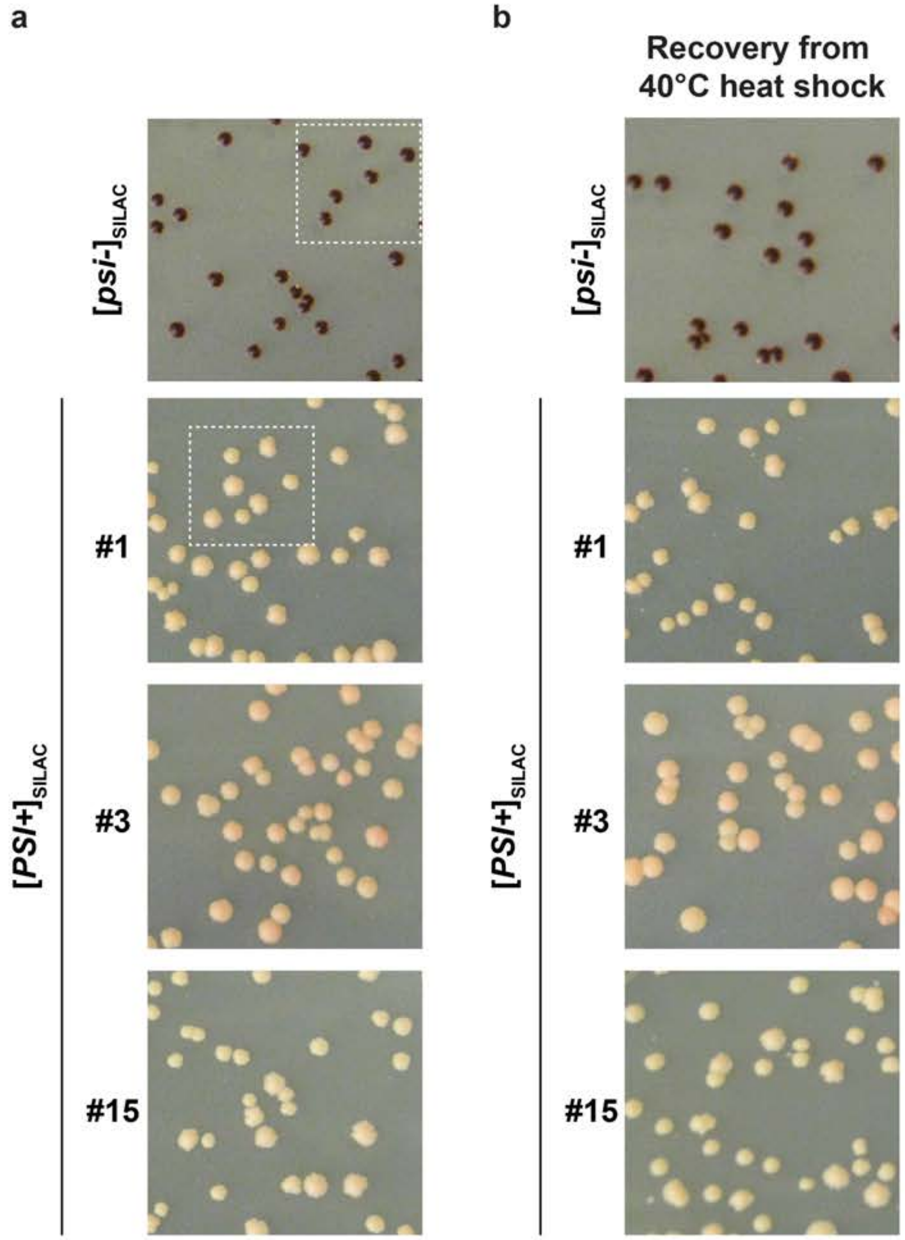

Figure S1. Stability of [PSI^+] in the SILAC strains.
a) [*psi*-]$_{\text{SILAC}}$, [*PSI*+]$_{\text{SILAC}}$ #1, 3, and 15 cells were plated on ¼ YPD media and grown at 30°C for several days. Insets of [*psi*-]$_{\text{SILAC}}$, [*PSI*+]$_{\text{SILAC}}$ #1 are shown in Figure 1d. **b)** Thermal stress [*PSI*+] stability assay. The indicated strains were first grown in log phase then plated on ¼ YPD media and incubated for 5 hours at 30˚C to ensure recovery, followed by exposure to thermal stress at 40˚C for 30 min. Strains were then grown at 30˚C for several days to assess for colony colour and sectoring.

Figure S2

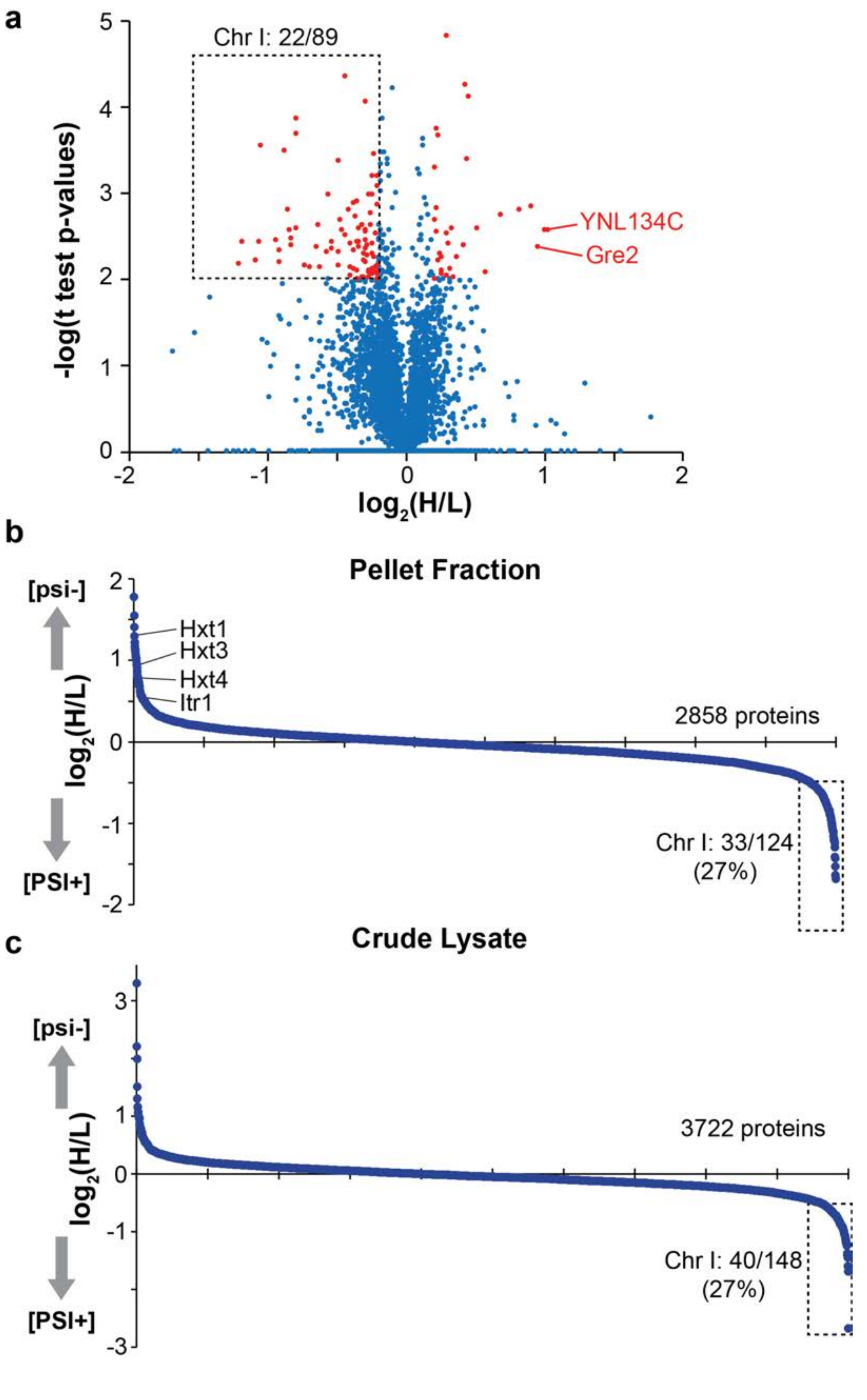

a
Chr I: 22/89
YNL134C
Gre2
-log(t test p-values)
log2(H/L)
b
Pellet Fraction
[psi-]
[PSI+]
Hxt1
Hxt3
Hxt4
Itr1
2858 proteins
Chr I: 33/124
(27%)
c
Crude Lysate
[psi-]
[PSI+]
3722 proteins
Chr I: 40/148
(27%)

Figure S2 (previous page). Proteome profiling of $[psi^-]_{SILAC}$ and $[PSI^+]_{SILAC}$ strains.

a) Volcano plot of protein quantified in the supernatant fraction derived from $[psi^-]_{SILAC}$ (heavy) versus $[PSI^+]_{SILAC}$ (light) cells. The x-axis shows the log_2(H/L) and y-axis corresponding log_{10}(p-values). In red are shown protein with log_2(H/L) < -0.2 or > 0.2 and log_{10}(p-values)> 2. **b and c)** Averaged log_2 ratios (H/L) of the proteins quantified in indicated samples ranked based on their ratio values. Ratios of the four designated hexose transporters in the pellet fraction are designated. The number of proteins expressed from chromosome I for proteins with log_2(H/L) < -0.5 are indicated.

Figure S3

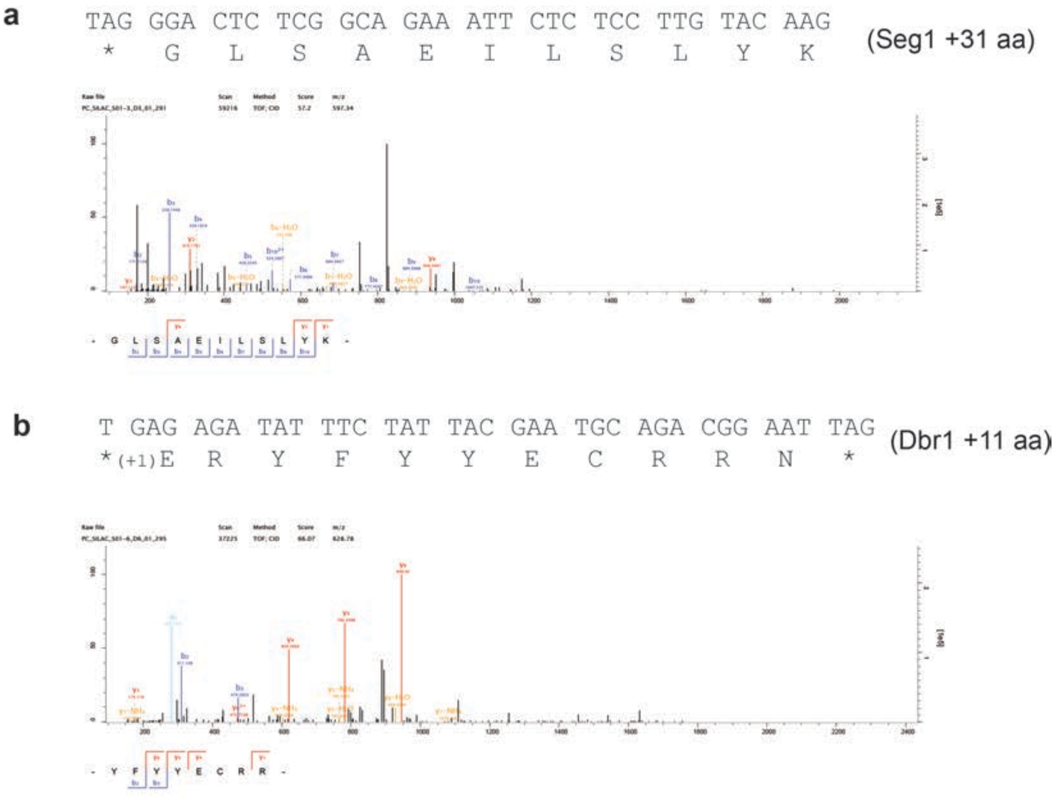

Figure S3. Spectra of peptides from read-through events

a and b) Two high confidence spectra matching possible C-terminal polypeptide extensions following translational read-though events were retained after manual validation of 98 candidate spectra. Data was first searched against the extended database in which proteins contained extended sequences after the stop codon in the three reading frames until the next stop codon. Candidate peptides against Seg1 and (a) and Dbr1 (b) were identified. Above the spectra, a short stretch of DNA sequence that starts with the stop codon and which overlaps with the identified peptide is shown.

Figure S4

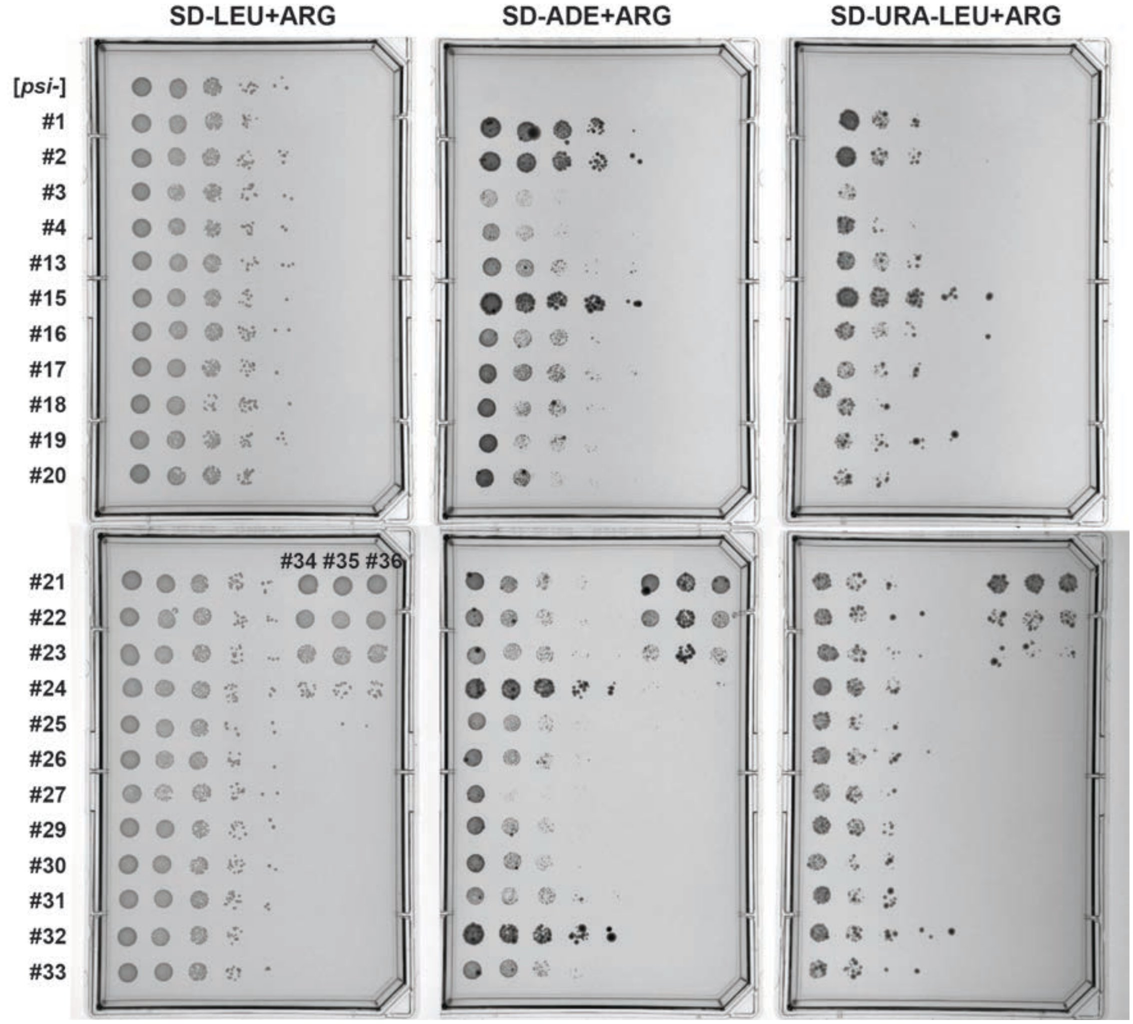

Figure S4. Characterizations of [PSI^{+}]$_{SILAC}$ strains.

The indicated [PSI^{+}]$_{SILAC}$ strains that carried out a *LEU2* plasmid with *ura3-14*were grown on the indicated synthetic defined (SD) plates containing 2% dextrose. Cropped images of this figure were used for Figure 3b.

Figure S5

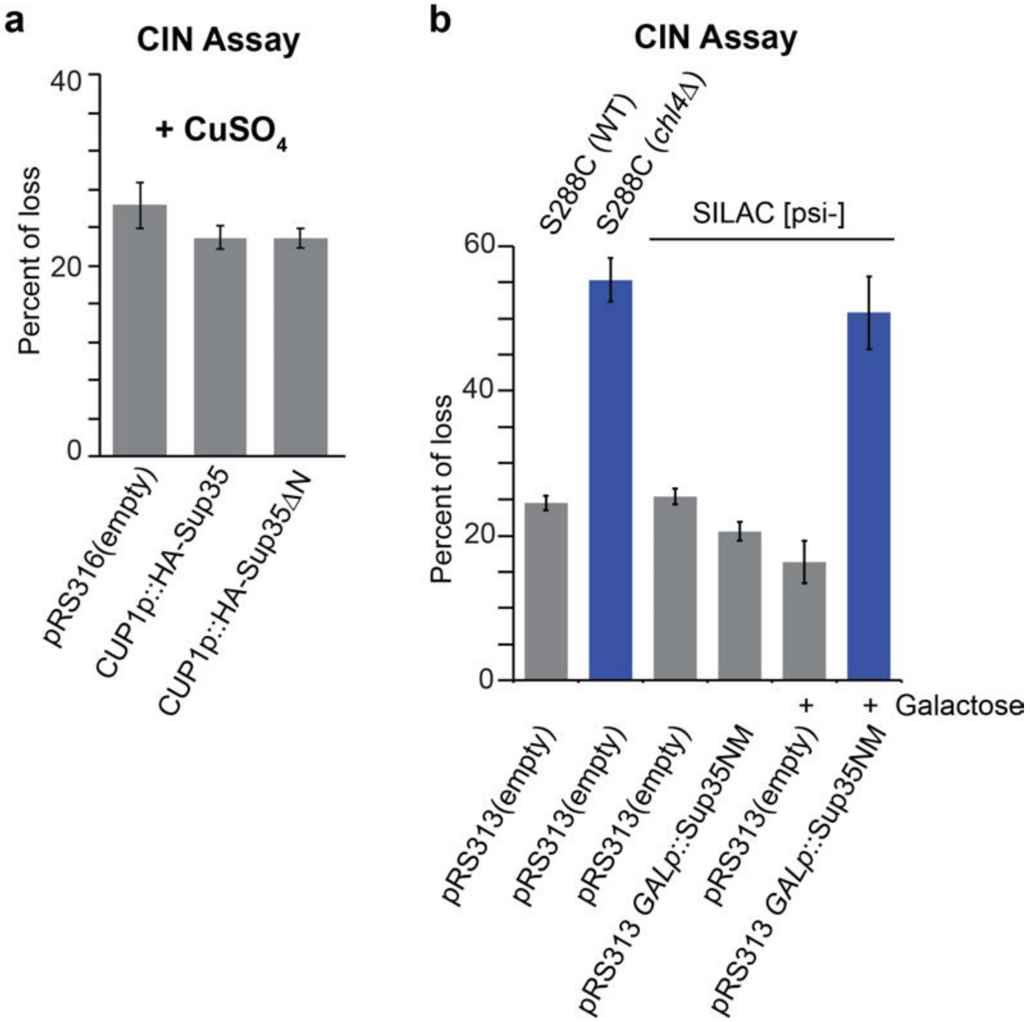

Figure S5. Chromosomal instability (CIN) upon expression of Sup35 or Sup35NM.
a) The loss of a *LEU2*-containing plasmid in S288C (BY4741) cells was assessed in presence of a pRS316 plasmid that carried out or not the full-length Sup35 controlled by the *CUP1* promoter or truncated Sup35 without its N-terminal domain. After incubation in SD-Ura-Leu with 100 µM $CuSO_4$ for 48 hours, cells were plated on SD-Ura and then replicated onto SD-Ura-Leu for 48 hours using 5 replicates. The percentage of *LEU2*-containing plasmid loss was determined by comparing the number of colonies on SD-Ura and SD-Ura-Leu plates: ((SD-Ura)-(SD-Ura-Leu))/(SD-Ura). **b)** Following incubation in YP-Raffinose media with or without galactose for 48 hours, the percentage of loss of the *HIS3*-containing plasmid was measured by comparing the number of colonies on SD+Arg-His and YPD plates: (YPD-SD)/YPD. In these experiments, the *HIS3*-containing plasmid was either empty or carried out the Sup35NM fragment under the *GAL* promoter.

Figure S6

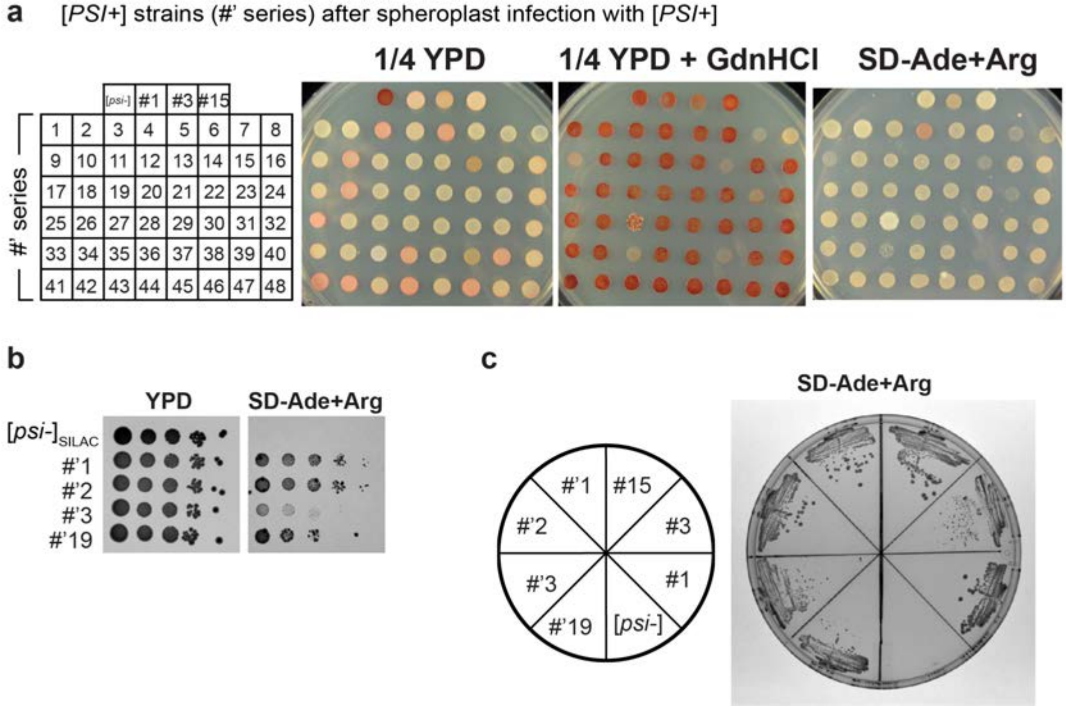

Figure S6. [PSI+] strains generated after spheroplast infection (#' series)

Assessing presence of chromosome (chr) I disomy in [*PSI+*] strains generated by infection with prion-containing yeast extract. **a)** 48 colonies picked from SD-Ade+Arg selection plate following [*PSI+*] yeast extract transformation were spotted on ¼ YPD with or without 4 mM GdnHCl, and SD-Ade+Arg. Strains were grown at 30˚C. **c)** Growth of indicated strains on YPD or SD-Ade+Arg, spotted at 1 O.D.$_{600}$ in 10-fold serial dilutions, then grown at 30˚C. **d)** Indicated strains from original series (right) and generated after spheroplast infection (left) were streaked on SD-Ade+Arg at 30˚C to compare growth of single colonies.

Figure S7

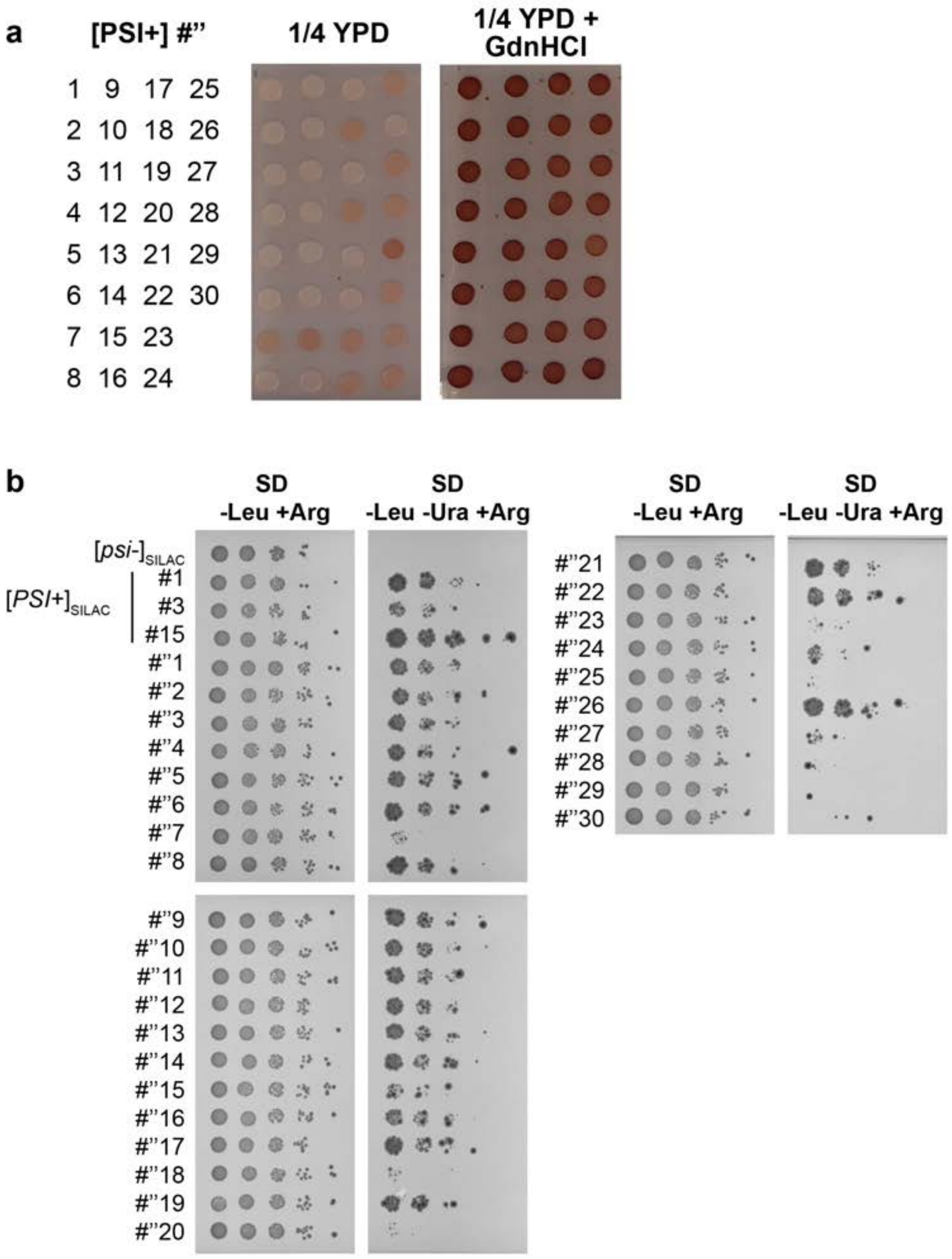

Figure S7. [PSI+] strains selected for NGS analysis (#'' series)

a) Thirty [*PSI*$^+$] strains were generated following Sup35NM overexpression in [*psi*$^-$]$_{SILAC}$ cells, but without growth selection in absence of adenine. The [*PSI*$^+$] strains were selected based on the coloration of the colonies. Reversibility of [*PSI*$^+$] was confirmed after plating the cells on ¼ YPD plate containing GdnHCl (right), in comparison to plating cells on ¼ YPD (middle). These strains are referred to [*PSI*$^+$] #'' followed by the indicated number (left) in the text. **b)** NGS [*PSI*+] strains #'' 1-30 selected on YPD were transformed with p*LEU2*-ura3-14 and spotted in 10-fold serial dilutions on SD-Leu+Arg or SD-Ura-Leu+Arg, with select SILAC strains for comparison. Strains were grown at 30˚C.

Supplementary Figure 8

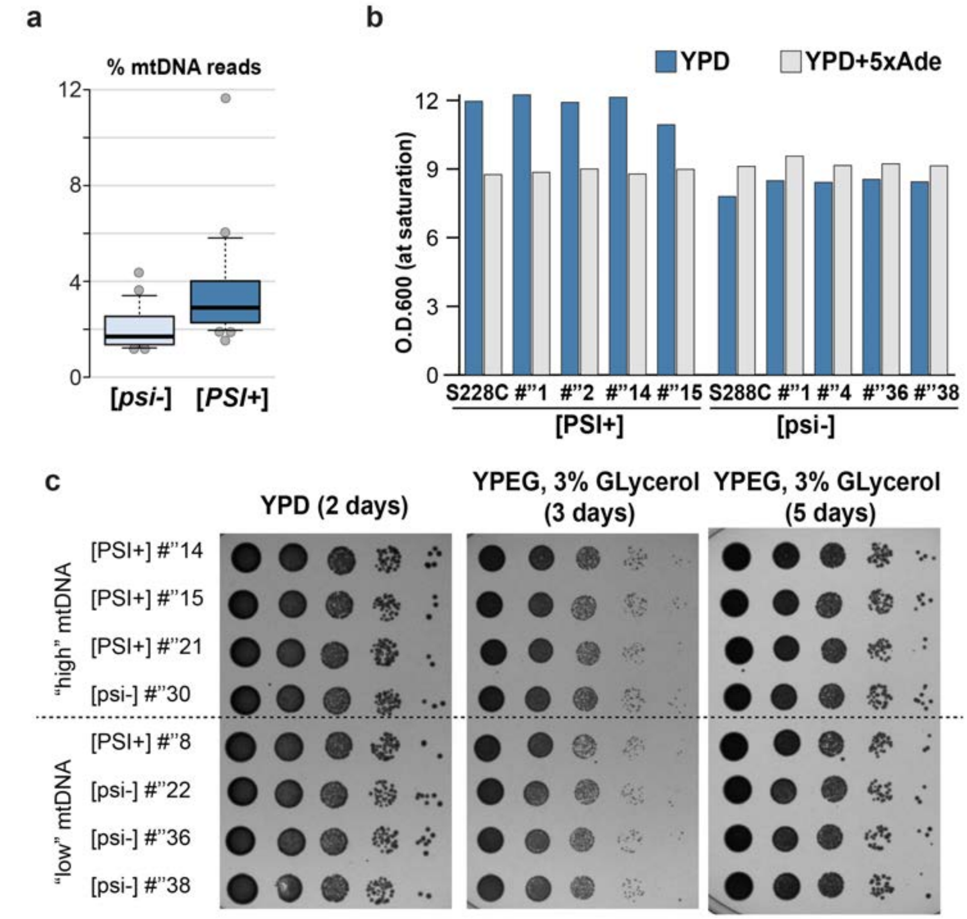

Figure S8. Analysis of mitochondrial DNA content

a) Box plot of percentage of sequencing reads matching mitochondrial (mt) DNA for the indicated 30 strains analyzed by NGS (thick bars indicate median values; box limits indicate the 25th and 75th percentiles). **b**) The O.D.$_{600}$ of cell cultures grown to saturation was measured for the indicated strains. Cell were either grown in YPD (blue) or in YPD supplemented with 100 mg / mL adenine (grey). **c)** Serial dilutions of the indicated strains on YPD or YPEG media that were grown for several days. The selected [PSI^+] and [psi^-] strains are representative of cells for which a high (top) or low (bottom) percentage of sequencing reads were matched to mtDNA. #'' designates strains generated for the NGS analysis that were selected on YPD plates (in presence of adenine) after the overexpression of Sup35NM.

Table S1. Table of strains

Strains	Genotypes	Database Entry
SILAC strain	BY4741, S288C, Mat a, *his3Δ1*, *leu2Δ0*, *ura3Δ0*, *MET15*, *arg4Δ::KanMX6*, *lys2Δ0*	YTM1173
[psi^-]	S288C, Mat α, [PIN^+], *his3Δ1*, *leu2Δ0*, *LYS2+*, *met15Δ0*, *ura3Δ0*, *can1Δ::STE2pr-SpHIS5*, *lyp1Δ::STE3pr-LEU2*, *ade1-14-cloNAT*	YTM1560
[PSI^+]	S288C, Mat α, [PIN^+], *his3Δ1*, *leu2Δ0*, *LYS2+*, *met15Δ0*, *ura3Δ0*, *can1Δ::STE2pr-SpHIS5*, *lyp1Δ::STE3pr-LEU2*, *ade1-14-cloNAT*	YTM1561
[psi^-]$_{SILAC}$	S288C, Mat α, [psi^-], [PIN^+], *his3Δ1*, *leu2Δ0*, *MET15*, *ura3Δ0*, *ade1-14::cloNAT*, *arg4Δ::KanMX6*, *lys2Δ0*	YTM1566
[psi^-]$_{SILAC}$	S288C, Mat α, [psi^-], [PIN^+], *his3Δ1*, *leu2Δ0*, *MET15*, *ura3Δ0*, *ade1-14::cloNAT*, *arg4Δ::KanMX6*, *lys2Δ0*, pRS313_GALp_Sup35NM_GFP	YTM1665
[PSI^+]$_{SILAC}$ #1	S288C, Mat α, [PSI^+], [PIN^+], *his3Δ1*, *leu2Δ0*, *MET15*, *ura3Δ0*, *ade1-14::cloNAT*, *arg4Δ::KanMX6*, *lys2Δ0*, pRS313_GALp_Sup35NM_GFP	YTM1711
[PSI^+]$_{SILAC}$ #2	S288C, Mat α, [PSI^+], [PIN^+], *his3Δ1*, *leu2Δ0*, *MET15*, *ura3Δ0*, *ade1-14::cloNAT*, *arg4Δ::KanMX6*, *lys2Δ0*, pRS313_GALp_Sup35NM_GFP	YTM1712
[PSI^+]$_{SILAC}$ #3	S288C, Mat α, [PSI^+], [PIN^+], *his3Δ1*, *leu2Δ0*, *MET15*, *ura3Δ0*, *ade1-14::cloNAT*, *arg4Δ::KanMX6*, *lys2Δ0*, pRS313_GALp_Sup35NM_GFP	YTM1713
[PSI^+]$_{SILAC}$ #4	S288C, Mat α, [PSI^+], [PIN^+], *his3Δ1*, *leu2Δ0*, *MET15*, *ura3Δ0*, *ade1-14::cloNAT*, *arg4Δ::KanMX6*, *lys2Δ0*, pRS313_GALp_Sup35NM_GFP	YTM1714
[PSI^+]$_{SILAC}$ #15	S288C, Mat α, [PSI^+], [PIN^+], *his3Δ1*, *leu2Δ0*, *MET15*, *ura3Δ0*, *ade1-14::cloNAT*, *arg4Δ::KanMX6*, *lys2Δ0*, pRS313_GALp_Sup35NM_GFP	YTM1644
[PSI^+]$_{SILAC}$ #23	S288C, Mat α, [PSI^+], [PIN^+], *his3Δ1*, *leu2Δ0*, *MET15*, *ura3Δ0*, *ade1-14::cloNAT*, *arg4Δ::KanMX6*, *lys2Δ0*, pRS313_GALp_Sup35NM_GFP	YTM1652
[PSI^+]$_{SILAC}$ #24	S288C, Mat α, [PSI^+], [PIN^+], *his3Δ1*, *leu2Δ0*, *MET15*, *ura3Δ0*, *ade1-14::cloNAT*, *arg4Δ::KanMX6*, *lys2Δ0*, pRS313_GALp_Sup35NM_GFP	YTM1653
[PSI^+]$_{SILAC}$ #30	S288C, Mat α, [PSI^+], [PIN^+], *his3Δ1*, *leu2Δ0*, *MET15*, *ura3Δ0*, *ade1-14::cloNAT*, *arg4Δ::KanMX6*, *lys2Δ0*, pRS313_GALp_Sup35NM_GFP	YTM1658
ctf19Δ	*Mat α, mfα1Δ::MFα1pr-LEU2 can1Δ::MFA1pr-HIS3 ura3Δ0 leu2Δ0 his3Δ1 lys2Δ0 ctf19Δ::natR*	YTM1619
chl4Δ	*Mat a;ura3-52 lys2-801 ade2-101 his3-Δ200 leu2-Δ1 trp1-Δ63 chl4Δ::kanMX6*	YTM1620

Table S2. Table of primers used for qPCR

Primer Name	Forward Primer (5'to3')	Reverse Primer (5' to 3')	Reference
Chr01La	ACAGCTTCTAAACGTTCCGTGTGC	GCGGTGTGTGGATGATGGTTTCAT	Pavelka et al., 2010
Chr01Ra	GCACTTGATCCATGTAGCCATACTCG	TTCGGGTGACCCTTATGGCATTCT	
Chr02La	TTTCAGGATCACGAGCGCCATCTA	CGGCAAGTGTCTCACTGTTGCATT	
Chr02Ra	ACGGTTGCACCGTTGTTCTTTCTG	CTGCGTGTGTTTCTGCGCGTTAAA	
ACT1	GTATGTGTAAAGCCGGTTTTG	CATGATACCTTGGTGTCTTGG	Taylor et al., 2005
COX1	CTACAGATACAGCATTTCCAAGA	GTGCCTGAATAGATGATAATGGT	

Supplementary Data (separate file)

Summary of the mass spectrometry analysis of three biological samples of [*PSI*$^+$] and [psi$^-$] cells that were labeled in light (L) and heavy (H) SILAC, respectively that was generated by MaxQuant. 8 samples were retained: three from each supernatant fraction (S01, S02, and S03); three from each crude lysate (TCL01, TCL02, and TCL03); and two from the pellet fractions (I01 and I02), as one the pellet sample was compromised during the processing and failed to provide high quality data. Only proteins identified with at least two peptides were retained. H/L ratios represents proteins abundance in [psi$^-$] versus [*PSI*$^+$] cells.
